# Tailoring a local acid-like microenvironment for efficient neutral hydrogen evolution

**DOI:** 10.1038/s41467-023-39963-8

**Published:** 2023-07-14

**Authors:** Xiaozhong Zheng, Xiaoyun Shi, Honghui Ning, Rui Yang, Bing Lu, Qian Luo, Shanjun Mao, Lingling Xi, Yong Wang

**Affiliations:** 1 Advanced Materials and Catalysis Group, Center of Chemistry for Frontier Technologies, State Key Laboratory of Clean Energy Utilization, Institute of Catalysis, Department of Chemistry, Zhejiang University, 310028 Hangzhou, P. R. China; 2 College of Chemistry and Molecular Engineering, Zhengzhou University, 450001 Zhengzhou, China

**Keywords:** Electrocatalysis, Hydrogen energy, Materials for energy and catalysis

## Abstract

Electrochemical hydrogen evolution reaction in neutral media is listed as the most difficult challenges of energy catalysis due to the sluggish kinetics. Herein, the Ir-H_x_WO_3_ catalyst is readily synthesized and exhibits enhanced performance for neutral hydrogen evolution reaction. H_x_WO_3_ support is functioned as proton sponge to create a local acid-like microenvironment around Ir metal sites by spontaneous injection of protons to WO_3_, as evidenced by spectroscopy and electrochemical analysis. Rationalize revitalized lattice-hydrogen species located in the interface are coupled with H_ad_ atoms on metallic Ir surfaces via thermodynamically favorable Volmer-Tafel steps, and thereby a fast kinetics. Elaborated Ir-H_x_WO_3_ demonstrates acid-like activity with a low overpotential of 20 mV at 10 mA cm^−2^ and low Tafel slope of 28 mV dec^−1^, which are even comparable to those in acidic environment. The concept exemplified in this work offer the possibilities for tailoring local reaction microenvironment to regulate catalytic activity and pathway.

## Abstract (web summary)

The development of neutral hydrogen evolution catalysts is challenging due to their sluggish kinetics. Here the authors report H_x_WO_3_ support which acts as proton sponge to create a local acid-like microenvironment around Ir sites, realizing acid-like hydrogen evolution rate in neutral media.

## Introduction

Sustainable electrocatalytic hydrogen evolution reaction (HER) using renewables powered, low-temperature water electrolyzers is promising for the deployment of the hydrogen economy for sustainable energy storage, transportation, and chemical production^1–7^. It is well-established that this reaction starts with the Volmer step, which generates adsorbed hydrogen intermediates (H_ad_) via electrochemical reduction of either hydronium ion (in an acidic medium) or water (in a neutral or alkaline medium). Subsequently, molecular hydrogen is produced by either a Tafel recombination step (H_ad_ + H_ad_ → * + H_2_) or a charge-transfer Heyrovsky step (H_ad_ + H_2_O + e^−^ → * + H_2_ + OH^−^)^8–13^. The kinetics of HER in neutral/alkaline medium is much sluggish than that in acidic environment because of the low concentration of protons. Consequently, even the state-of-the-art Pt-based catalyst, shows two to three orders of magnitude lower activity in neutral/alkaline media as compared to acidic media^14^. The kinetics of HER is strongly relevant to both the nature of electrode materials and the local reaction microenvironment in the vicinity of the catalytic sites at electrolyte–solid interface^15–20^. To date, substantial progress has been made in promoting the kinetics of the electrode reactions by ameliorating the catalyst materials through various ways, such as introducing oxophilic active elements^21,22^, heterostructure modulating^23,24^, strain engineering^25,26^, nanoscopic confinement^27,28^. Apparently, in most cases, these conventional strategies can only modulate their electronic states, adsorption capability of intermediates, and thereby catalytic properties in a gradual or mild way. However, these still cannot get rid of the pH-dependent kinetics of HER, leading to the inability to achieve big breakthroughs in the non-acidic electrolyte. Therefore, selecting a suitable system to create a local acid-like environment through multiple physicochemical effects to the maximum extent possible, will provide an alternative way to promote the electrocatalytic performance and guide the higher efficiency electrocatalyst design in non-acidic electrolyte, especially in more challenging neutral media.

As the prototypical example, the reversible and rapid hydrogen doping of WO_3_ to form H_*x*_WO_3_ bronze was well reported via electrochemical electron–proton co-doping^29–33^, in which H atoms are incorporated as W-OH species with Brønsted acidity and reducing some W^6+^ to W^5+^ along with charge rearrangement. Protonated H_x_WO_3_ could act as a proton sponge and electron reservoir to create an acid-like microenvironment in the electric double layer, thereby further affecting the reaction barriers and pathway^34–36^. However, the strong adsorption of hydrogen by basic oxygen centers and surface frustrated H–H coupling process severely hinder the local acidic species being rationally utilized. Enhancing electrochemical activation capability of H_x_WO_3_ via the addition of cocatalysts is of interest^37–40^^.^ Up to now, it still encounters many problems and challenges, for example, (1) the degree of local acidification of H_x_WO_3_ in neutral media has not been accurately quantified; (2) the synergistic catalysis between local acidic species and co-catalysts has not been fully understood; (3) the enhanced activity cannot be simply attributed to a single optimized catalytic site, and the origin of the activity deserves further investigation. In this work, typical tungsten oxide nanorods-arrays aligned on carbon fibers were deliberately selected as a host material. The WO_3_ support experienced hydrogen insertion and Ir nanoparticles (NPs) electrodeposition processes to form hybridized Ir–H_*x*_WO_3_. Experimental results and theoretical calculations deciphered that a high density of surface revitalized WO–H species enrich local proton concentration around Ir sites, and act as scavengers for H_ad_ on the metallic Ir to realize expedited hydrogen evolution rate, which is reflected in the fact that Ir–H_*x*_WO_3_ exhibits acid-like HER properties in neutral media.

## Results and discussion

### Hydrogen intercalation and storage of WO_3_

**Fig. 1:**
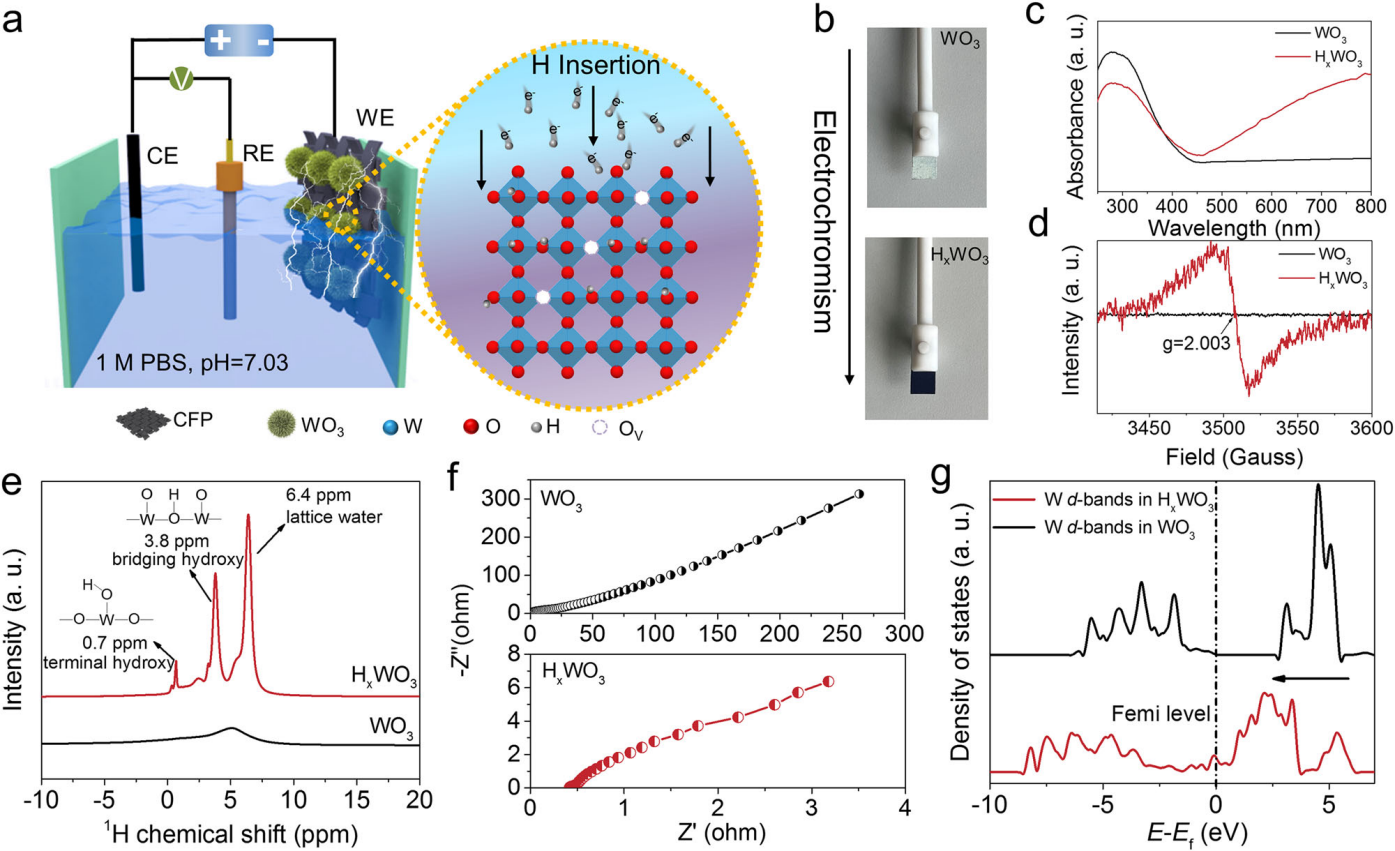
Hydrogen intercalation of WO_3_. **a** Schematic illustration of the hydrogen intercalation process of WO_3_ to form H_*x*_WO_3._ **b** Digital pictures of the WO_3_ working electrode before and after electrochemical hydrogenation. **c** UV–visible diffuse reflectance spectra of WO_3_ and H_*x*_WO_3._ **d** EPR signal of oxygen vacancies. **e** ^1^H NMR spectra of WO_3_ and H_*x*_WO_3_. **f** EIS Nyquist plots of different electrocatalysts measured at open circuit potential from 10 kHz to 0.01 Hz. **g** The pDOS curves of W *d*-bands of WO_3_ and H_*x*_WO_3_.

The formation mechanism of H_x_WO_3_ bronze is dependent on the double injection of electrons and protons to the WO_3_ nanorods grown on carbon fiber paper under cyclic voltammetry measurement (between 0 and −0.35 V vs. reversible hydrogen electrode, RHE) in 1.0 M PBS, as shown in Fig. 1a and Supplementary Fig. 1. Obviously, the color of the WO_3_ electrode turns from yellow to dark blue (Fig. 1b and Supplementary Movie 1), which is a typical electrochromic process. Correspondingly, UV–vis diffuse reflectance spectra exhibit a prominent increase of absorption at a wavelength above 450 nm after electrochemical hydrogenation (Fig. 1c), typically resulting from light absorption of the free electrons at oxygen vacancies (O_v_) and the d-d transition of W^5+^–OH (refs. ^31,41^). With increasing negative potentials, the electrons accumulated at the electrode/electrolyte interface, thus attracting the protons to reduce the lattice oxygen to produce O_v_ via the following reaction equation^31^, as reflected by a significant elctron paramagnetic resonance (EPR) signal at *g* = 2.003 in Fig. 1d.

1 $${{{\mbox{W}}}}^{6+}{{{\mbox{O}}}}_{3}+2{{{\mbox{e}}}}^{-}+{{{\mbox{H}}}}^{+}\to {{{\mbox{W}}}}^{5+}{{{\mbox{O}}}}_{2}-{{{\mbox{O}}}}_{{{\mbox{V}}}}+{{\mbox{O}}}{{{\mbox{H}}}}^{-}$$

Surface active O_v_ is conductive to adsorbing and subsequently inducing dissociation of water molecules via transferring a proton to adjacent oxygen, thus forming terminal and bridging hydroxyls groups (W^5+^-–OH species) via the following reaction equation:

2 $${{{\mbox{W}}}}^{5+}{{{\mbox{O}}}}_{2}-{{{\mbox{O}}}}_{{{\mbox{V}}}}+{{{\mbox{H}}}}_{2}{{\mbox{O}}}\to {{{\mbox{W}}}}^{5+}{{{\mbox{O}}}}_{2}{{\mbox{H}}}-{{\mbox{OH}}}$$

Undoubtedly, ^1^H solid-state nuclear magnetic resonance (NMR) spectroscopy (Fig. 1e) and X-ray photoelectron spectroscopy (XPS) results (Supplementary Fig. 2) confirm the above statements. Hydrogen intercalation fundamentally changes the interface properties of WO_3_. Electrochemical impedance spectroscopy (EIS, Fig. 1f) and Mott–Schottky measurement (M–S, Supplementary Fig. 3) are highly sensitive to detect the interface electron transfer and carrier density^42,43^, respectively. An interface transfer resistance and corresponding d*C*^−2^*/*d*V* significantly decreased after electrochemical protonation, which is ascribed to the high conductivity of W^5+^–OH polaron states and high mobility of hydrogen on the WO_3_ surface. Furthermore, protonation-driven semiconductor–metal conversion of WO_3_ arises from a shift in electronic structure that is describable as a repositioning of the W *d*-bands in Fig. 1g.

### Local pH measurements and catalytic behaviors of H_*x*_WO_3_

**Fig. 2:**
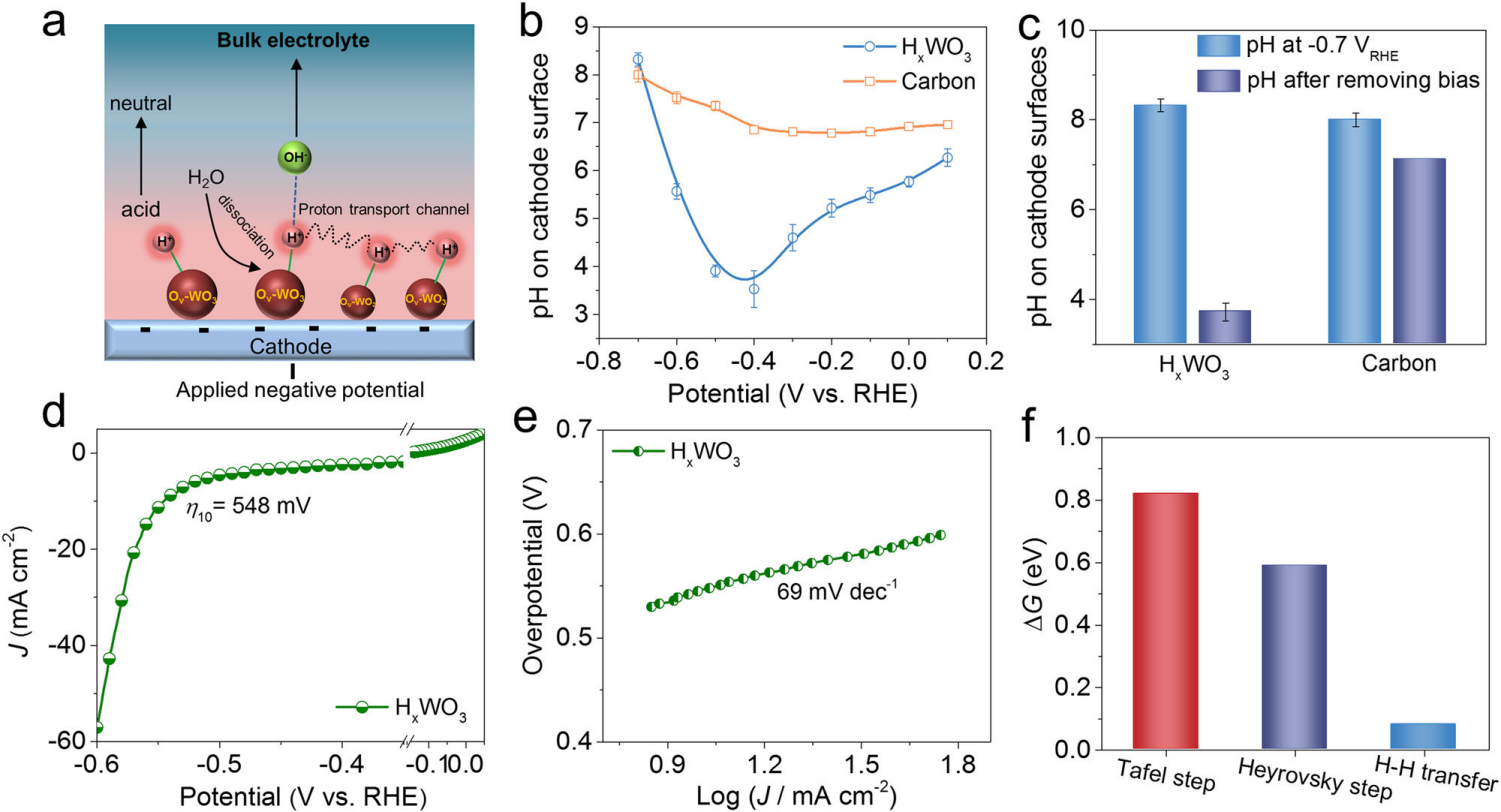
Local pH measurements and catalytic behaviors of H_x_WO_3_. **a** Schematic diagram of local acid-like microenvironment generation on H_*x*_WO_3_ cathode. **b** Measured pH values on H_*x*_WO_3_ and carbon cathode surfaces at different potentials, respectively. **c** The pH on H_*x*_WO_3_ and carbon cathode surfaces before and after removing bias (−0.7 V_RHE_). **d** HER polarization curves of H_*x*_WO_3_ grown on CFP with 95% iR compensation and (**e**) corresponding Tafel plots. **f** Free energy barriers of H–H coupling and H–H transfer in H_*x*_WO_3_. Note: error bars represent the standard deviation of three independent measurements.

We used the rotating ring-disk electrode (RRDE) technique^19,44,45^ to quantitatively detect local pH on the H_x_WO_3_ cathode surfaces at different applied potentials in neutral 1.0 M PBS solution (bulk pH is 7.03), along with classical carbon support for comparison (Supplementary Fig. 4–6, details see Methods). On the basis of RRDE detecting method, we found that the pH of the H_*x*_WO_3_ surface varies from 6.27 to 3.53 as potential decreases from 0.1 to −0.4 V_RHE_, which undoubtedly confirms that H_x_WO_3_ acts as proton sponge to form a local acid-like microenvironment on the electrode surface (Fig. 2a, b). In sharp contrast, the pH of carbon cathode surface is maintained at ~7 in the range of 0.1 to −0.4 V_RHE_ (Fig. 2b). As the potential continued to shift negatively, the pH of H_*x*_WO_3_ and carbon support surfaces gradually increases and approaches 8.32 and 8.0 at −0.7 V_RHE_, respectively, due to the consumption of the local hydrogen species. Astonishingly, when the bias (−0.7 V_RHE_) is removed, the surface of H_x_WO_3_ and carbon cathodes turn back to steady acidic (pH = 3.73) and neutral (pH = 7.12) states, respectively (Fig. 2c). We then move on to investigate the catalytic behaviors of H_x_WO_3_ in neutral media. Counterintuitively, despite the construction of a unique proton-rich microenvironment, the H_x_WO_3_ grown on CFP material still provides a high overpotential of 548 mV at 10 mA cm^−2^ (Fig. 2d). Following the insertion of hydrogen atoms, H-H coupling must occur to form molecular H_2_. This can take place through surface-mediated (Tafel step) or water-mediated (Heyrovsky step) routes^8^. In the surface-mediated mechanism, it is hard for two surface hydrogen atoms of H_x_WO_3_ to proceed H–H coupling because active hydrides are separated by relatively long distances (Supplementary Fig. 7), which is further reflected by high Tafel barrier (0.82 eV in Fig. 2f). In the water-mediated mechanism, surface hydrogen atoms form H_2_ by reacting with water of the electrolyte. The analysis of the Tafel slope for H_x_WO_3_ (Fig. 1e) reveals that it obeys the water-mediated mechanism, but requires a high overpotential (>500 mV) to drive it. As shown by density functional theory (DFT) calculations in Fig. 2f and Supplementary Fig. 8, the poor catalytic activity of H_*x*_WO_3_ catalyst is well explained by the fact that surface water-mediated H-H transfer predominates in the early stage of HER and water-mediated H-H coupling occurs only when the overpotential is largely increased. The weak electrochemical activation capability of H_x_WO_3_ severely prevents its hydrides from being properly utilized^46^. To tackle this problem, the construction of H_x_WO_3_ with metal-based materials is considered as a promising design strategy in the following two aspects: (1) interfacial polarization electric field generated by metal and H_*x*_WO_3_ to activate lattice-hydrogen species; (2) providing additional active sites to promote interfacial synergistic effects between them.

### Synthesis and characterization of catalysts

**Fig. 3:**
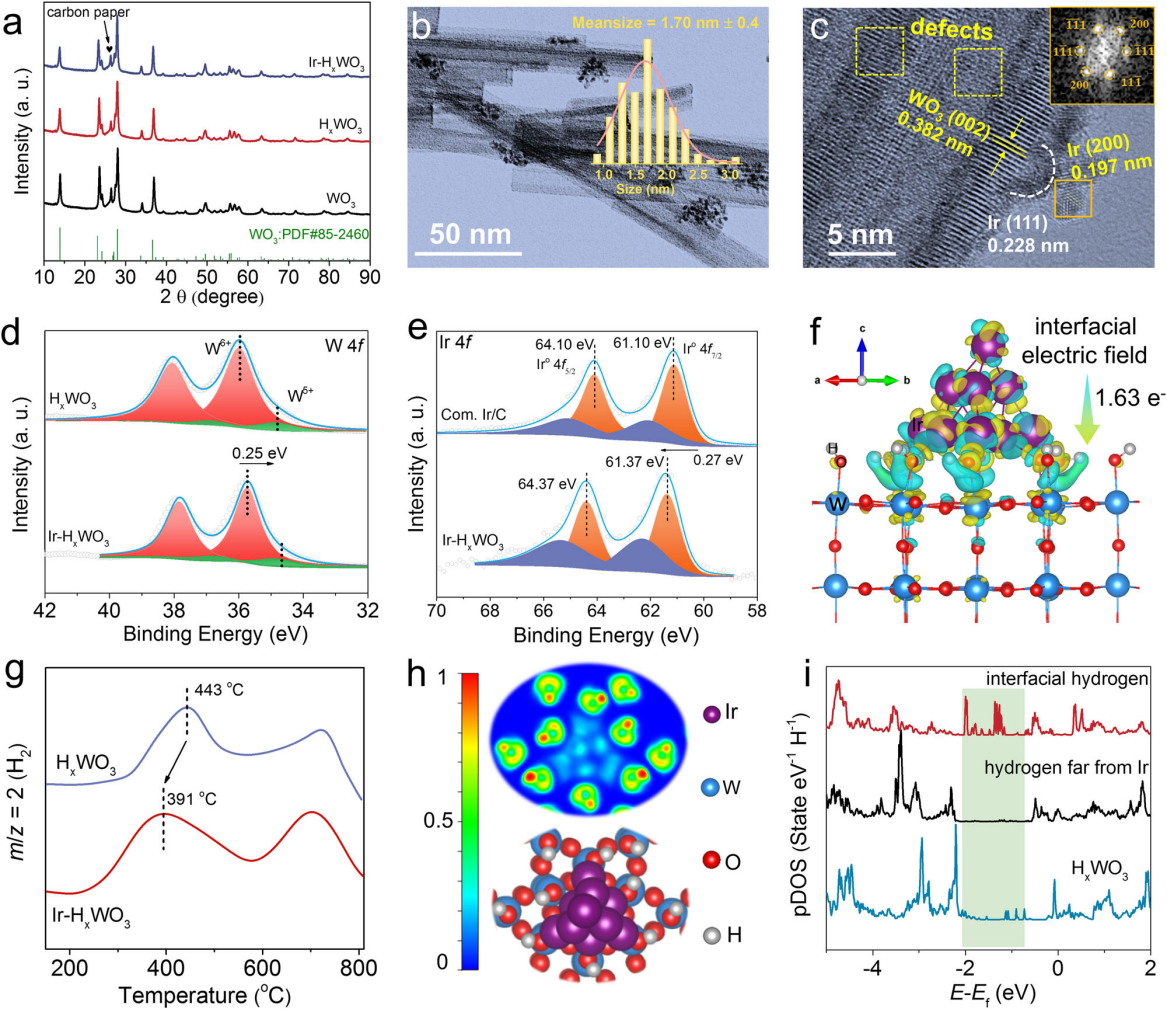
Structural characteristics of catalysts. **a** XRD patterns of as-obtained WO_3_, H_*x*_WO_3_ and Ir–H_*x*_WO_3_. **b** Typical TEM image of Ir–H_*x*_WO_3_, the inert in (**b**) shows the Ir NPs size distribution pattern. **c** HAADF-STEM images of Ir–H_*x*_WO_3_, with the inset in (**c**) giving the fast Fourier transform of the corresponding Ir NP. **d** High-resolution W 4*f* spectra of Ir–H_*x*_WO_3_ and H_*x*_WO_3_. **e** High-resolution Ir 4*f* spectra of Ir–H_*x*_WO_3_ and commercial Ir/C. **f** The side views of charge density difference in the interface of Ir_10_ clusters supported on H_*x*_WO_3_. The cyan region reflects an electron-deficient state while the yellow region reflects an electron-rich area, with an isovalue of 0.004 e^–^/Å^3^. **g** Representative TPD-MS thermal desorption profiles for Ir–H_*x*_WO_3_ and H_*x*_WO_3_ and the main desorbed specie detected is H_2_ with *m*/*e* = 2. **h** Electron localization function evaluations of Ir_10_-H_x_WO_3_. **i** The pDOS curves of 1*s* orbitals of different hydrogen atoms in Ir_10_–H_*x*_WO_3_ and pure H_*x*_WO_3_.

Based on the above assumptions, Ir–H_*x*_WO_3_ hybrid electrocatalyst was intentionally manufactured similar to that of H_x_WO_3_ except for adding 0.2 g L^−1^ IrCl_3_ to 1.0 M PBS solution. Ir^3+^ precursors are progressively reduced to metallic Ir nanoparticles (NPs) under applied negative bias, as revealed by the HER activity enhancement increases with cycles, eventually leveling off after 500 cycles in Supplementary Fig. 9. Ir content in Ir–H_*x*_WO_3_ hybrid electrocatalyst is 2.8 wt% (or 47 μg cm^−2^ when normalized to geometric area of electrode) determined by inductively coupled plasma-optical emission spectrometry (ICP-OES, Supplementary Table 1). X-ray diffraction (XRD) patterns in Fig. 3a disclose that as-prepared WO_3_, H_*x*_WO_3_, and Ir–H_*x*_WO_3_ electrocatalysts exhibit clear diffraction peaks of hexagonal WO_3_ (JCPDS No. 85-2460), but no diffraction peaks related to H_*x*_WO_3_. Notably, the characteristic diffraction peaks of WO_3_ in H_*x*_WO_3_ and Ir–H_*x*_WO_3_ catalysts are shifted toward a low-angle direction (Supplementary Fig. 10), probably ascribing to oxygen defect-induced lattice extension^47^. Meanwhile, for Ir–H_*x*_WO_3,_ the signals of Ir NPs are not detected, possibly due to their small sizes and low content. As revealed in Supplementary Fig. 11, 12 and Fig. 13a–c, after electrochemical protonation, the scanning electron microscopy (SEM) and transmission electron microscopy (TEM) images display that H_x_WO_3_ and Ir–H_*x*_WO_3_ samples inherit the original WO_3_ nanorods-like morphology. Comparatively, the TEM images (Fig. 3b and Supplementary Fig. 13d–f) of Ir–H_*x*_WO_3_ demonstrate that Ir NPs with mean size of ~1.7 nm are embedded on H_*x*_WO_3_ nanorods. Corresponding EDS elemental mapping images and line-scan profiles (Supplementary Fig. 13g–k) further verify the successful loading of Ir. The Ir NPs exhibit clear lattice fringes with interplanar distances of 1.97 and 2.28 Å, corresponding to the [200] and [111] crystal planes of the [011] face-centered cubic Ir, respectively, further evidenced by the fast Fourier transform (FFT) pattern (Fig. 3c). Meanwhile, the disordered structures identified by deformed and blurred lattice fringes belonging to [002] plane of WO_3_ are found in Ir–H_*x*_WO_3_ (Fig. 3c), which is ascribed to a good deal of surface defects induced by hydrogen intercalation during electrochemical activation^48^. Subsequently, XPS was carried out to further analyze the atomic structure and surface valence of catalysts. The typical W 4*f* spectra of H_x_WO_3_ and Ir–H_*x*_WO_3_ indicate the coexistence of W^6+^ and W^5+^ (Fig. 3d)^30^. Compared with those of H_x_WO_3_, W 4*f* binding energies of the Ir–H_*x*_WO_3_ show a significant redshift of 0.24 eV. The O 1*s* split-peak fitting results (Supplementary Fig. 14) reveal that lattice oxygen, surface hydroxyl, and adsorbed water species are detected on the surface of H_*x*_WO_3_ and Ir–H_*x*_WO_3_ samples. As excepted, after Ir NPs loading, the O 1*s* spectrum of Ir–H_*x*_WO_3_ moves toward lower binding energies. Figure 3e demonstrates the comparison of Ir 4*f* XPS spectra between commercial Ir/C and Ir–H_*x*_WO_3_. It is noticeable that the binding energy of Ir for Ir–H_*x*_WO_3_ is positively shifted by 0.27 eV up to 61.37 (Ir^o^ 4*f*_7/2_) and 64.37 eV (Ir^o^ 4*f*_5/2_). These results manifested the strong electronic interaction between Ir NPs and H_*x*_WO_3_ support. Further, the charge density difference diagram of the interface between Ir NPs and H_*x*_WO_3_ (Fig. 3f) reveals a charge transfer of 1.63 e^-^ from the Ir atom to H_x_WO_3_ through interfacial Ir-O-W bond, confirming the XPS results. In addition, the planar average potential plots along the *Z*-direction (Supplementary Fig. 15) confirm the interfacial built-in electric fields in Ir–H_*x*_WO_3._

Undoubtedly, the strong electric field effect at the interface between Ir NPs and H_*x*_WO_3_ will affect the lattice-hydrogen species in H_*x*_WO_3_ support. The ^1^H NMR spectra (Supplementary Fig. 16) confirm the signals of Ir–H_*x*_WO_3_ broadens and moves toward higher fields (1.1 and 4.0 ppm) compared to those of H_*x*_WO_3_ (0.7 and 3.8 ppm), which indisputably reveal that the acid strength of Ir–H_*x*_WO_3_ material is stronger than that of H_*x*_WO_3_ (ref. ^49^). Stronger acid strength also means easier detachment of protons. To prove this statement, temperature-programmed desorption (TPD) coupled with mass spectrometry (*m/z* = 2, H_2_) was performed to investigate the desorption behavior of lattice-hydrogen species over H_*x*_WO_3_ and Ir–H_*x*_WO_3_. As demonstrated in Fig. 3g, the TPD profiles of Ir–H_*x*_WO_3_ and H_*x*_WO_3_ are characterized by two typical peaks, assigned to the recombination of surface and bulk hydroxyl groups producing H_2_ (ref. ^50^), respectively. It is worth noting that the introduction of Ir accelerates the desorption of lattice-hydrogen species in the H_x_WO_3_ support, as revealed in shifting towards low temperature of Ir–H_*x*_WO_3_ TPD pattern compared to pure H_*x*_WO_3_. We next considered the chemical reasons for why Ir metal profoundly changes the behavior of hydrogen desorption over H_*x*_WO_3_ surfaces. The contour map of electron localization function (ELF) for Ir–H_*x*_WO_3_ is shown in Fig. 3h, projected over the [001] plane to analyze the electron localization and bond polarity characters^51^. The addition of Ir in the H_*x*_WO_3_ structure leads to the localized electron density redistribution of WO–H species adjacent to Ir NPs (denoted as interfacial hydrogen species) and the formation of more polar O–H bonds. Contrariwise, O–H bonds far from Ir metal exhibit a considerably covalent character. Bader charge analysis was conducted to quantitatively study the charge distribution. In Supplementary Fig. 17, the average Bader charges value of interfacial hydrogen atoms is 0.341 e^−^, which is significantly lower than that of the hydrogen atoms far away from Ir (0.365 e^−^) and pure H_x_WO_3_ (0.361 e^−^). Further extracting the projected density of states (pDOS) curves of different chemical states of lattice-hydrogen species, as shown in Fig. 3i, it is well noted that interfacial-hydrogen species possess a higher electronic state near the Fermi level compared to pure H_*x*_WO_3_ and hydrogen species far from Ir metal, indicating that metallic Ir revitalizes the lattice-hydrogen species and enabling it possible to participate in the HER process.

### Electrocatalytic HER performances

**Fig. 4:**
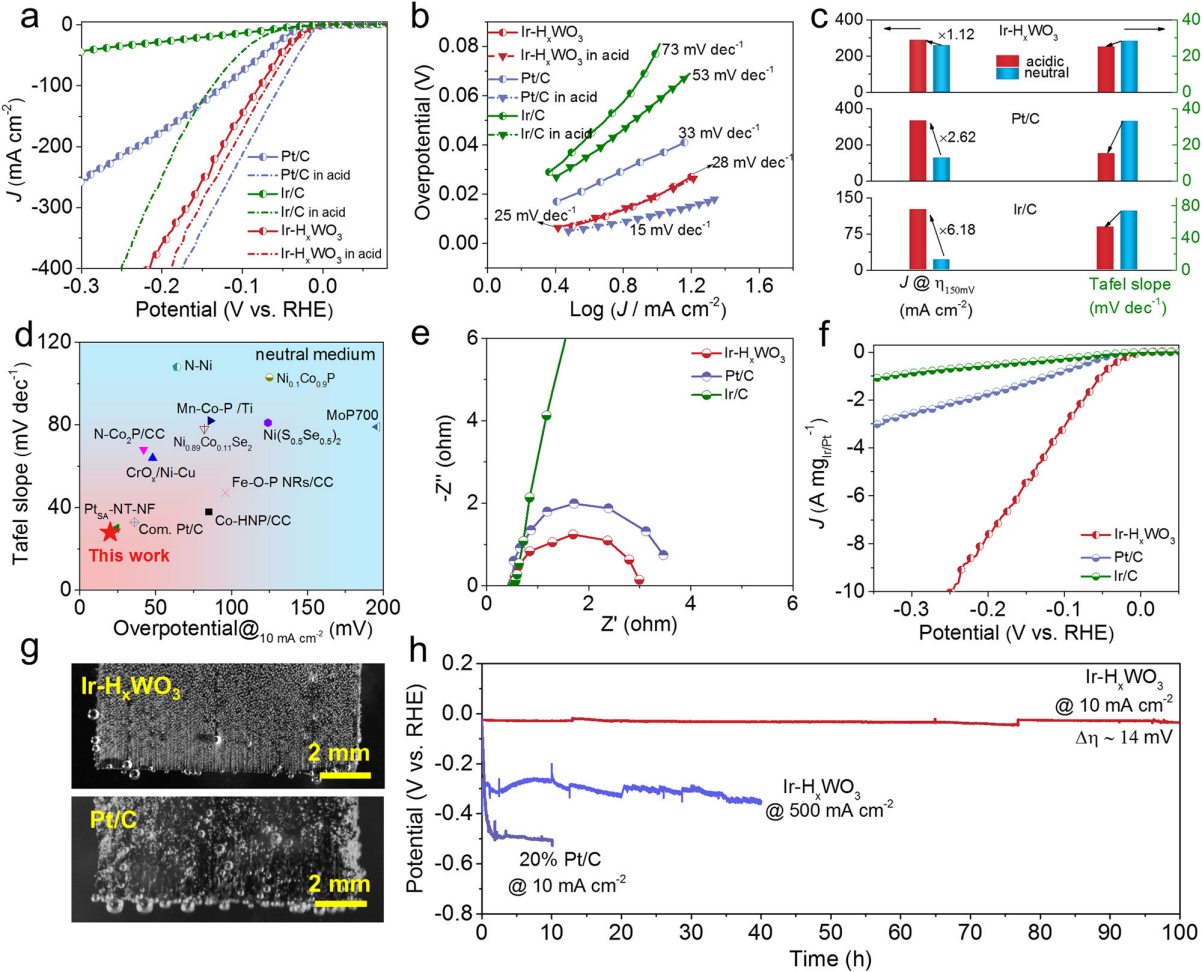
Electrocatalytic HER performances. **a** HER polarization curves of different electrocatalysts in 1.0 M PBS and 0.5 M H_2_SO_4_ at 2 mV s^−1^ scan rate. **b** Tafel plots obtained from the polarization curves. **c** Comparison of current density (@η_150 mV_) and Tafel slopes of different electrocatalysts in acidic and neutral media. **d** Comparison of neutral HER activity between Ir–H_*x*_WO_3_ and recently reported electrocatalysts. **e** EIS Nyquist plots of different electrocatalysts measured at −0.03 V_RHE_ from 10 kHz to 0.01 Hz in 1.0 M PBS. **f** Mass activity of electrocatalysts. **g** The H_2_ bubbles desorption behaviors of Ir–H_*x*_WO_3_ and commercial Pt/C at 100 mA cm^−2^. **h** Chronopotentiometry tests of Ir–H_*x*_WO_3_ (@10 and 500 mA cm^−2^) and commercial 20% Pt/C (@10 mA cm^−2^) in 1.0 M PBS. All electrochemical data were corrected for 95% iR drop.

Encouraged by a local activated acid-like microenvironment created around Ir NPs in Ir–H_*x*_WO_3_, it is expected to be able to substantially boost HER activity in non-acidic environments. Thus, the electrocatalytic HER activity of Ir–H_*x*_WO_3_, commercial 20 wt% Pt/C and 10 wt% Ir/C catalysts were tested in a conventional three-electrode electrochemical cell with H_2_-saturated 1.0 M PBS as electrolyte (Supplementary Fig. 18), along with acidic HER evaluation in 0.5 M H_2_SO_4_ for comparison. The catalytic activity was obtained from iR-compensated linear sweep voltammetry (LSV) curves, and different levels of iR compensation were considered and presented in Supplementary Fig. 19. To avoid potentiostat oscillation and overcorrected results^52^, automatically 95% iR compensation was adopted in this electrochemical test. As expected, in Fig. 4a–c, the HER activity and kinetics of Ir–H_*x*_WO_3_ under neutral conditions are similar to those in acidic media. Specifically, at an overpotential of 150 mV, Ir–H_*x*_WO_3_ can deliver 256 mA cm^−2^ in neutral media, but it only increases to 277 mA cm^−2^ under acidic media. The close Tafel slope values (neutral: 28 mV dec^−1^; acidic: 25 mV dec^−1^) indicate a similar hydrogen evolution pathway (Tafel step: H_ad_ + H_ad_ → * + H_2_). However, striking performance discrepancy is observed in well-known Pt/C and Ir/C electrocatalysts in different electrolytes. In acidic media, the current density of Pt/C and Ir/C is 2.62 and 6.18 times higher than those in neutral medium at 150 mV overpotential. The significantly reduced Tafel slopes manifests that HER catalytic kinetics is strongly related to the proton concentration. These results corroborate that for conventional carbon-supported metal catalysts exhibit highly pH-dependent catalytic activity, while H_*x*_WO_3_ plays as proton sponge to create acid-like microenvironment around metal catalysts to obtain thermodynamically favorable catalytic activity and accelerated reaction kinetics in a proton-deficient neutral media. It is noteworthy that the Ir–H_*x*_WO_3_ (η_10_ = 20 mV; Tafel slope: 28 mV dec^−1^) is among the best HER electrocatalysts reported in the neutral medium (Fig. 4d and Supplementary Table 2). Accelerated HER kinetic of Ir–H_*x*_WO_3_ is also reflected by the smaller charge-transfer resistance (the width of the semicircle, 2.5 Ω, Fig. 4e). To further identify its high intrinsic HER activity, being normalized to the per milligram noble-metal loading, Ir–H_*x*_WO_3_ (5.46 A mg_Ir_^−1^) exhibits 4.3 and 13.3 times higher mass activity than those of Pt/C (1.27 A mg_Pt_^−1^) and Ir/C (0.41 A mg_Ir_^−1^) at an overpotential of 150 mV (Fig. 4f).

High-speed imaging experiments prove that the superhydrophilic structure (surface W–OH group) of Ir–H_*x*_WO_3_ better suppresses bubble coalescence and enhances bubble release compared to commercial Pt/C counterpart (Fig. 4g and Supplementary Movie 2–3), so Ir–H_*x*_WO_3_ was proposed to have better catalytic performance for water splitting, especially at high current densities. Additionally, we performed the stability test for fully assessing the catalyst via the accelerating degradation technique (ADT) and potential-constant electrolysis. Supplementary Fig. 20 shows the polarization curve of Ir–H_*x*_WO_3_ manifests negligible shift after 10,000 ADT cycling tests. The long-term durability testing on the Ir–H_*x*_WO_3_ catalyst by a static chronopotentiometry test (at 10 mA cm^−2^ in Fig. 4h) even represents a relatively stable horizontal line with an overpotential increase of only 14 mV over 100 operating hours, while Pt/C exhibits a clipping activity decay within a few hours, demonstrating the good activity retention of Ir–H_*x*_WO_3_. However, it is still a challenge to develop efficient catalysts working at large current density for neutral water splitting. Amazingly, Ir–H_*x*_WO_3_ catalyst maintains operation stability at a large current density of 500 mA cm^−2^ over 40 h, outperforming most of the recently reported landmark catalysts (Supplementary Table 2). Both the structure and composition of Ir–H_*x*_WO_3_ remain unchanged before and after the HER stability test as examined by XRD, SEM, HRTEM and ICP-OES (Supplementary Fig. 21 and Table 2). For practical application, natural seawater splitting performance is also evaluated. As expected, in Supplementary Fig. 22, Ir–H_*x*_WO_3_ exhibits good HER performance in natural seawater, requiring an overpotential of 150 mV to produce 10 mA cm^−2^ of catalytic current density. This is lower than that of Pt/C (295 mV) and other state-of-art catalysts (Supplementary Table 3). In summary, the efficient Ir–H_*x*_WO_3_ catalyst under neutral/near-neutral conditions is expected to be applied for next-generation water-splitting technologies.

### Exploration of catalytic mechanisms

**Fig. 5:**
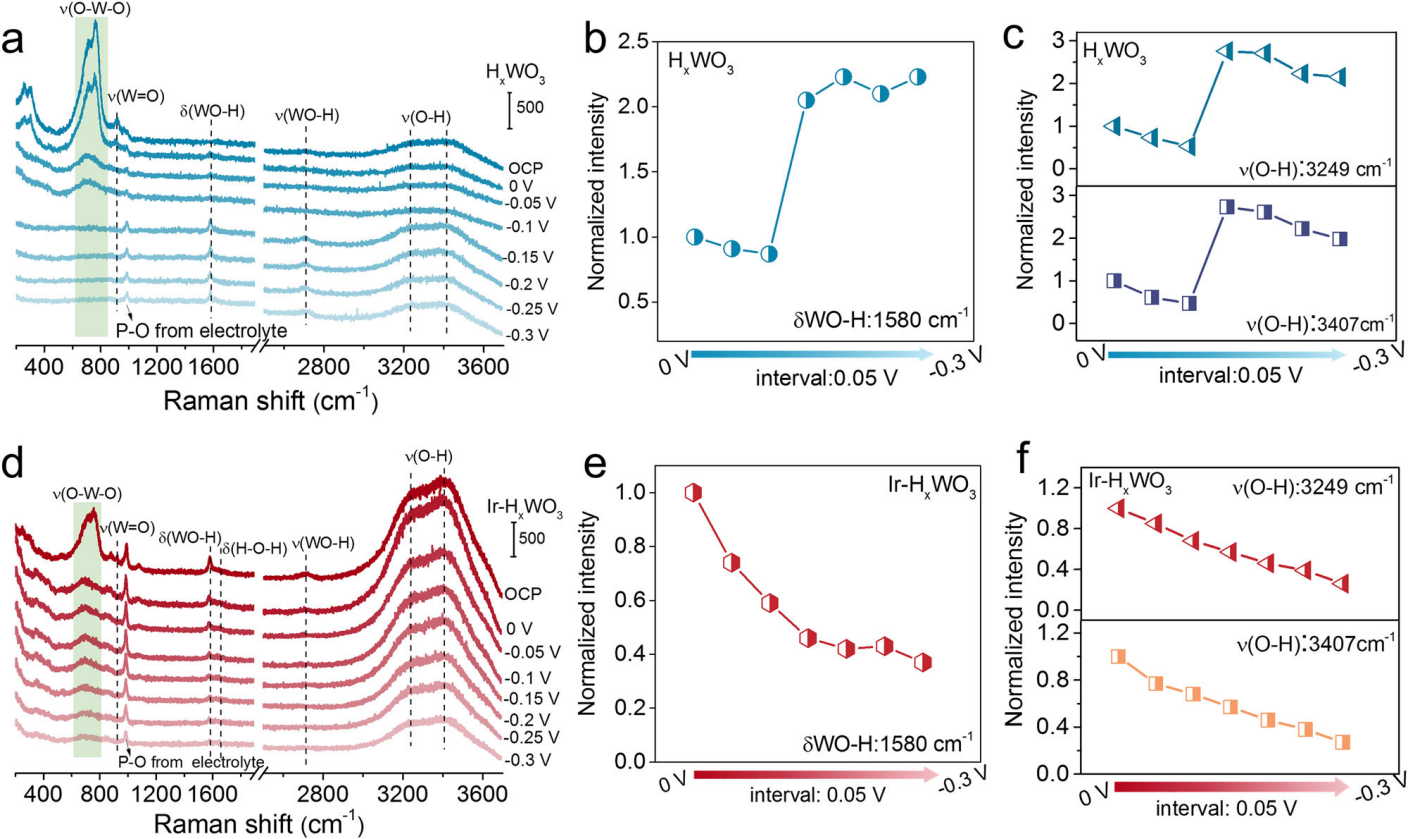
Operando electrochemical Raman measurements. **a** Operando electrochemical Raman spectra of H_*x*_WO_3_ from OCP to −0.3 V_RHE_ and corresponding normalized intensity of WO–H (**b**) and O–H (**c**) signals under different HER potentials. **d** Operando electrochemical Raman spectra of Ir–H_*x*_WO_3_ from OCP to −0.3 V_RHE_ and corresponding normalized intensity of WO–H (**e**) and O–H (**f**) signals under different HER potentials.

To gain more insights into the original active sites of electrocatalysts, the operando electrochemical Raman spectra were then recorded to investigate the neutral HER behavior at the Ir–H_*x*_WO_3_ and H_*x*_WO_3_ surfaces (Supplementary Fig. 23). For H_*x*_WO_3_ sample, as indicated in Fig. 5a, b, the intensity of typical peaks at 762 cm^−1^, 714 cm^−1^ (stretching vibrations of O–W–O skeleton) and 926 cm^−1^ (stretching vibrations of terminal W = O bond) sharply weaken from open circuit potential (OCP) to −0.1 V_RHE._ Further decreasing potential (−0.15 to −0.3 V_RHE_), all of these characteristic peaks disappear, accompanied by the increasing intensity of the ∼1580 and 2715 cm^−1^ band from the bending and stretching vibration modes of WO–H species^53–55^. This observation confirms that hydrogen insertion occurs in WO_3_ in response to the cathodic voltage, where the H atoms are incorporated into the Brønsted acidic W^5+^–OH groups, and the hydrogen storage saturates after the potential reaches −0.15 V_RHE_. However, the lack of H–H coupling sites and weak electrochemical activation prevent the surface hydride from being effective as H_2_ molecular from the surface, which is reflected by its poor catalytic activity. Additionally, the variation trends of the two broad bands at 3249 and 3407 cm^−1^ (Fig. 5a, c), assignable to tetrahedrally and trihedrally coordinated water at the interface^56,57^, suggest that the process of hydrogen incorporation is closely related to the activation of interfacial water. In stark contrast, with regard to Ir-modified H_*x*_WO_3_ electrocatalyst (Fig. 5d, e), the Raman signals of W–O–W, W = O and WO–H progressively weaken over the whole potential range (OCP to −0.3 V_RHE_), which further illustrates that the Ir–H_*x*_WO_3_ depleted surface WO–H species during HER process to alleviate deep-hydrogenation of H_*x*_WO_3_ supports. Moreover, compared with H_*x*_WO_3_, the apparent increased intensity of O–H stretching vibrations signals at 3249 and 3407 cm^−1^ and the appearance of H–O–H bending vibration bands at 1650 cm^−1^ are detected^57^ (Fig. 5d, f), indicating that Ir NPs enhance the adsorption of interfacial water molecules. As subsequent HER going on, the activity of H_2_O continued to decrease, verifying that Ir serves as the site of water dissociation. Based on the results of operando electrochemical Raman spectra, it is plausible that there may be possible synergistic catalysis between Ir and lattice-hydrogen species derived from H_x_WO_3_.

**Fig. 6:**
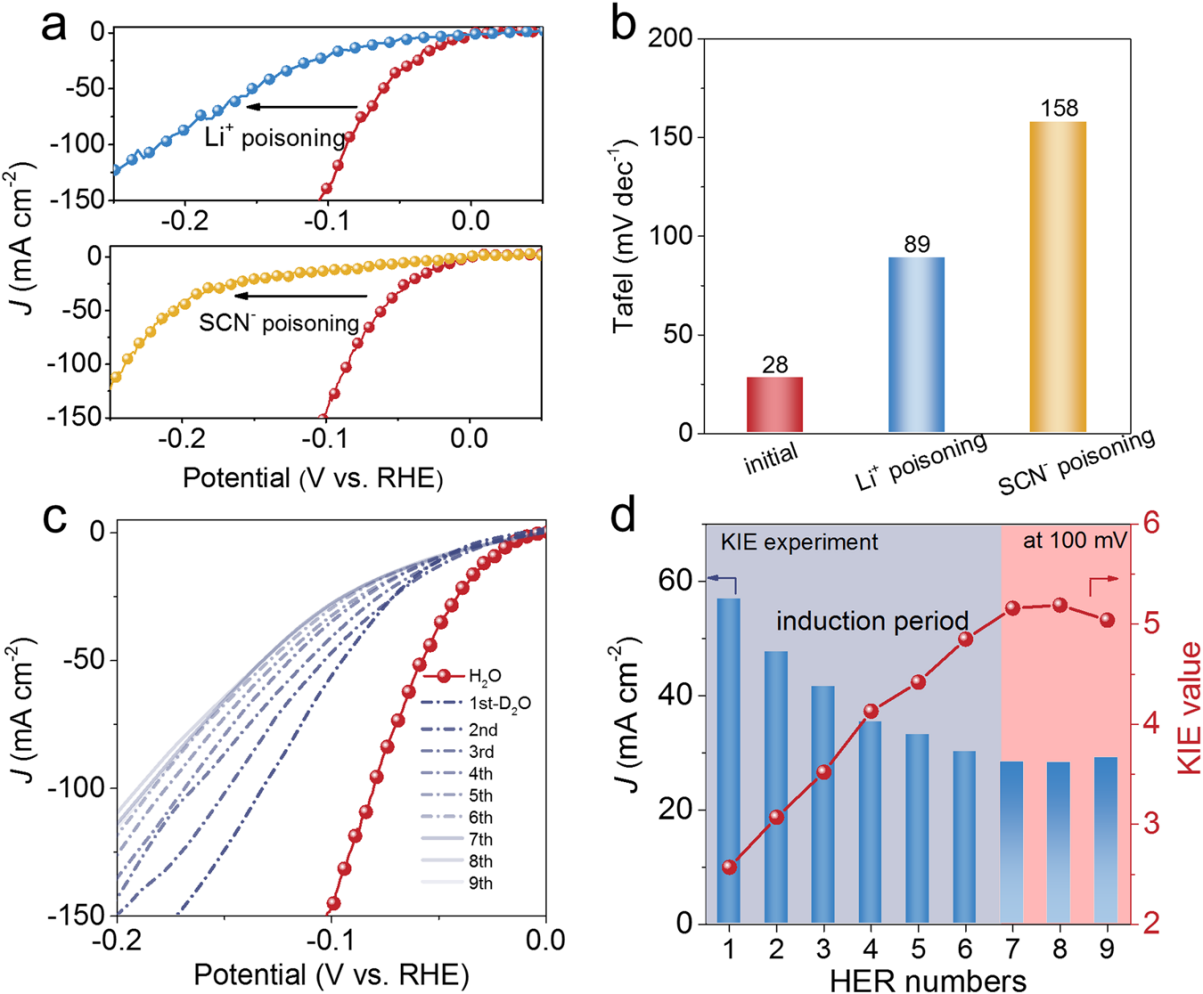
Poisoning and KIE experiments. **a** The LSV curves of Ir–H_*x*_WO_3_ before and after poisoning metallic Ir sites and WO–H sites, respectively. **b** The Tafel slope comparisons of Ir–H_*x*_WO_3_ before and after poisoning experiments. **c** The LSV curves of Ir–H_*x*_WO_3_ in 1.0 M PBS (H_2_O and D_2_O) electrolyte. **d** Calculated KIE values (JH_2_O/JD_2_O) under 100 mV overpotential. All electrochemical data were corrected for 95% iR drop.

To verify this hypothesis, first of all, selectively poisoning experiments were conducted. Based on the well-established Li^+^-ion-exchange method^35^ (WO–H + Li^+^ → WO-Li + H^+^), Ir–H_*x*_WO_3_ electrocatalyst was immersed in 0.5 M LiNO_3_ solution for 12 h to completely realize H^+^–Li^+^ exchange. As shown in Fig. 6a, after Li^+^ poisoning, the striking activity decay of Ir–H_*x*_WO_3_ is observed, highlighting the importance of the local acidified environment for HER activity. The same process was performed on Pt/C and H_x_WO_3_ support in Supplementary Fig. 24 for comparison to confirm Li^+^ selectively poisons the WO–H species and excludes its effect on the metal sites. In addition, it is well known that metal catalytic sites are very sensitive and reactive to SCN^−^, which can poison metal-centered catalytic sites by coordinating with metallic species strongly^58^. The HER polarization plots of Ir–H_*x*_WO_3_ show the drastic negative shifts of potentials after the introduction of 20 mM SCN^−^ ions into the electrolyte (Fig. 6a). For comparison, after SCN^-^ treatments for Pt/C and H_x_WO_3_ support in Supplementary Fig. 25, an obvious activity reduction of Pt/C is detected and has no effect on H_x_WO_3_ support. Then, the Tafel slopes of Ir–H_*x*_WO_3_ catalyst before and after Li^+^ or SCN^-^ poisoning are compared to analyze the change of the catalytic reaction pathway. As shown in Fig. 6b and Supplementary Fig. 26, after WO–H replaced by WO–Li, the value of Tafel slope of catalyst increases from 28 to 89 mV dec^−1^, manifesting that the rate-determining step (RDS) of the reaction changes from Tafel (H_ad_ + H_ad_ → * + H_2_) to Heyrovsky step (H_ad_ + H_2_O + e^−^ → * + H_2_ + OH^−^)^12^ and elucidating that the presence of lattice-hydrogen alters the catalytic mechanism. Furthermore, after poisoning the metal sites, the RDS transforms into Volmer step (158 mV dec^−1^, *H_2_O + e^−^ → *H + OH^−^)^12^, significantly unveiling that Ir metal site is the response for the water dissociation, which is in line with the results of operando Raman spectra.

Further depicting the rate-limiting step, kinetic isotope effect (KIE) experiments were performed. The LSV curves and corresponding KIE values (*J*H_2_O/*J*D_2_O at −0.1 V_RHE_) are recorded from Ir–H_*x*_WO_3_ in 1.0 M PBS aqueous electrolyte and 1.0 M PBS D_2_O electrolyte (Fig. 6c, d). Interestingly, the LSV plots of Ir–H_*x*_WO_3_ demonstrate the negative shifts of potentials after several HER cycles and eventually stabilize after 7th LSV scan. These results reveal that the initial KIE effect (2.57) is possibly derived from the O–D bond activation/dissociation of deuterium water, and then surface active WO–H species are gradually replaced by WO–D, resulting in worse hydrogen transfer kinetics^59^, which can be explained by the presence of an obvious induction period and corresponding increased KIE value up to 5.16. To further confirm the KIE effect, the pre-synthesized Ir–D_*x*_WO_3_ (see Methods) also manifests a similar induction period in 1.0 M PBS (H_2_O) electrolyte, showing enhanced HER activity (Supplementary Fig. 27). The same process was carried out for Pt/C in Supplementary Fig. 28 for comparison. Pt/C only exhibits the typical isotope effect (KIE = 3.79 at −0.1 V_RHE_) and does not have the above-mentioned induction period phenomenon. Compared with Pt/C, the large KIE value variations of Ir–H_*x*_WO_3_ (2.57_initial_ < 3.79_Pt/C_ < 5.16_final_) indicate the unsubstitutable role of lattice-hydrogen species in the HER process. Combining operando Raman measurements, selective poisoning and kinetic isotope effect experiments, it unambiguously unveiled that a potential neutral HER mechanism involved lattice WO–H species and metallic Ir site synergistic catalysis pathway in Ir–H_*x*_WO_3_.

**Fig. 7:**
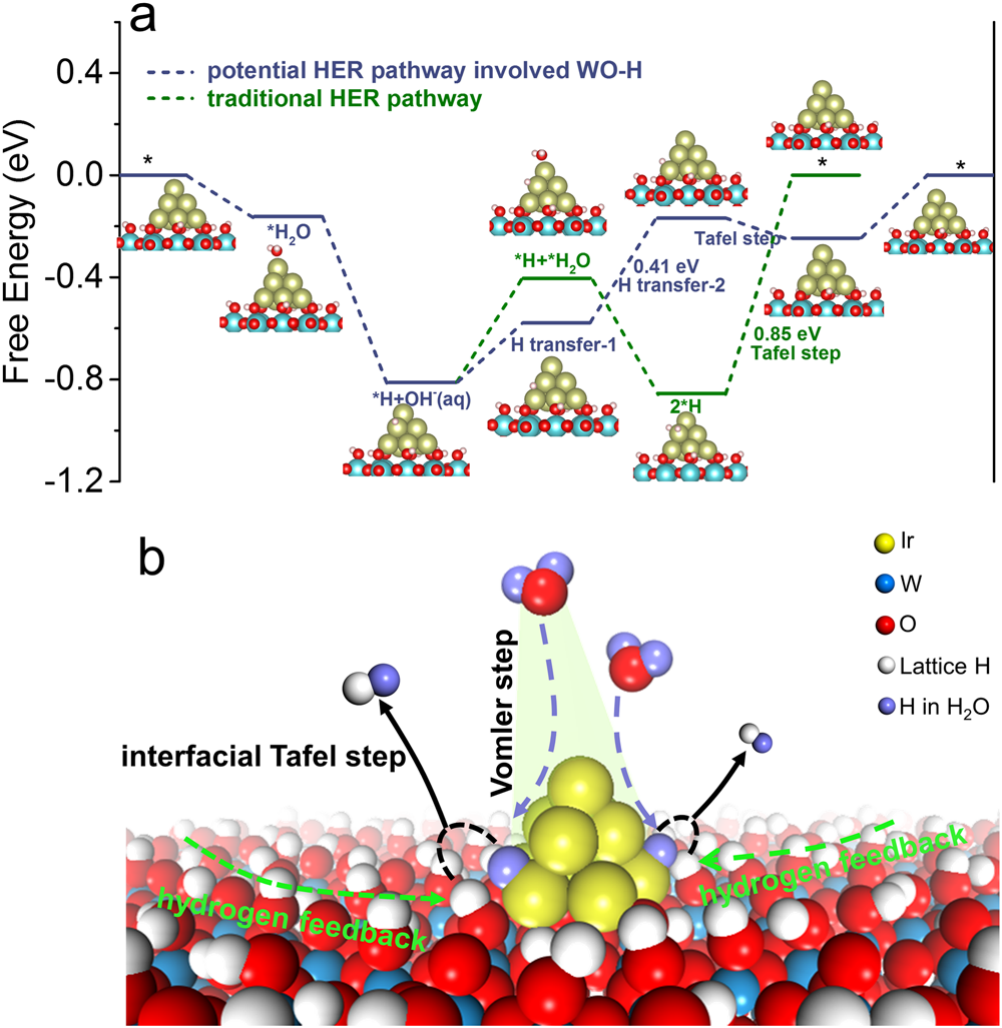
DFT calculations and catalytic mechanisms. **a** Free energy profiles for HER over Ir_10_–H_*x*_WO_3_ via different reaction pathways. **b** Schematic illustrating the proposed reaction mechanism on Ir–H_*x*_WO_3_.

Density functional theory (DFT) calculations were then performed to probe the specific reaction pathway of Ir–H_*x*_WO_3_. Based on the above catalyst characterization, the computational model adopt Ir_10_ cluster supported on WO_3_ (002) with terminal oxygen saturated with hydrogen atoms to form experimentally validated WO–H species. The possible HER pathway assisted by support-derived lattice-hydrogen species (WO–H) is systematically investigated in comparison to the traditional HER process (Fig. 7a). The adsorption and dissociation of the H_2_O molecule were first examined. The absorbed free energy of H_2_O at different Ir sites (Ir_1_, Ir_2_, and Ir_3_ correspond to the interfacial Ir, subinterfacial Ir and bulk Ir, respectively) are calculated (Supplementary Fig. 29). Clearly, Ir_3_ active site is found to be the most favorable (or dominant) site for H_2_O adsorption with more negative Δ*G*_H2O*_ (−0.16 eV) as compared with that of Ir_1_ (0.29 eV) and Ir_2_ (0.12 eV). Subsequently, *H_2_O dissociation is exothermic by 0.66 eV, and the formed H atom is adsorbed on the neighboring Ir_2_ site. The Tafel slope for Ir–H_*x*_WO_3_ suggests that hydrogen evolution over this material should occur via recombination of two H atoms. Then, two Tafel pathways are considered: (1) Traditional Tafel pathway (green line). Another H_2_O is adsorbed on Ir_3_ and cleaved to *H and hydroxyl; two H atoms on Ir_2_ site are preferentially coupled together by Tafel reaction with a high free energy barrier (0.85 eV). (2) Interfacial Tafel pathway involved lattice-hydrogen (violet line). *H on the Ir_2_ site undergoes two-steps hydrogen transfer to the Ir_1_ site with a substantially low energy barriers (0.41 eV), subsequent Tafel step for Ir_1_–*H and adjacent WO–*H species experiences an exothermic process (−0.08 eV), which contributes to fast hydrogen production rate, as obtained experimentally. Moreover, this unique and dominant interfacial dehydrogenation site is further corroborated by H_2_-TPD experiments in Supplementary Fig. 30.

Based on the conclusions obtained from the above experimental and theoretical studies, we propose a possible interfacial hydrogen-evolution pathway mediated by lattice hydrogen of neutral HER catalyzed by hybridized Ir–H_*x*_WO_3_ (Fig. 7b). The Ir metal component in the hybridized Ir–H_*x*_WO_3_ possesses superior electrocatalytic activity toward the Volmer process and is expediently utilized to strongly adsorb H_2_O, effectively catalyze the dissociation of H_2_O* to generate interfacial Ir–*H. The thermodynamically favorable Tafel process is advantageously utilized to efficiently combine the interfacial Ir–H* and neighboring reactive WO–H* into H_2_. The hydrogen-deficient state at the interface can be eliminated by the hydrogen transfer on the surface of H_x_WO_3_ to replenish hydrogen, thereby realizing the closed loop of the entire catalytic reaction. Additionally, Supplementary Movies 4 and 5 visually demonstrate the continuous replenishment of hydrogen on Ir–H_*x*_WO_3_ surface after removing bias, along with Pt/C for comparison. Hence, the exceptional neutral HER electrocatalytic performance of Ir–H_*x*_WO_3_ stems from the coherent synergism of Ir and in situ formed lattice-hydrogen components. Inspired by the local acid-like microenvironment created by H_*x*_WO_3_, it is expected to extend “proton sponge” effect of H_x_WO_3_ support to other *M*–H_*x*_WO_3_ systems (*M* = Pt, Ru, Pd, Co, Ni). As expected in Supplementary Fig. 31, compared with conventional *M*-Carbon catalysts, the significantly reduced overpotentials and accelerated reaction kinetics on *M*–H_*x*_WO_3_ systems (*M* = Ru, Pt, Pd, Co, Ni) confirm that the local acid-like microenvironment provided by H_x_WO_3_ fundamentally enhances the intrinsic HER activity of catalysts in thermodynamically unfavorable neutral media.

### Neutral water electrolysis device performance

**Fig. 8:**
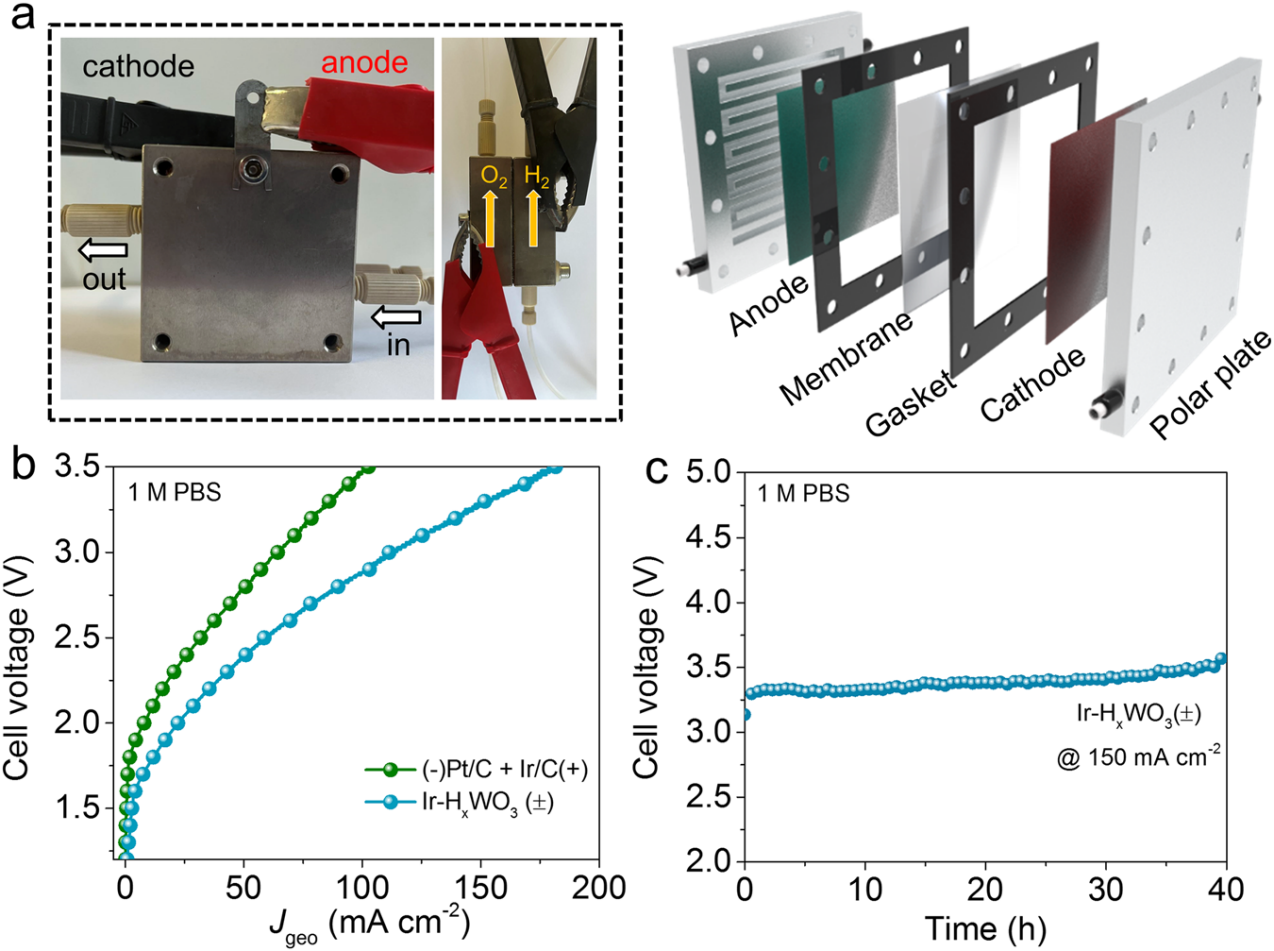
Neutral water electrolysis device performance. **a** Photographs and schematic illustration of membrane electrode assembly (MEA) electrochemical reactor, the geometric area of the electrode is 4 cm^2^. **b** Neutral water splitting performance of the commercial (−)Pt/C + Ir/C(+) and Ir–H_*x*_WO_3_/CFP(±) MEA setups at room temperature without iR compensation. **c** Stability tests of the Ir–H_*x*_WO_3_/CFP(±) MEA.

To highlight the practical significance of localized acidification environmental engineering for neutral water reduction, we further integrated bifunctional Ir–H_*x*_WO_3_ catalysts into a membrane electrode assembly (MEA) as cathode and anode materials and assembled an actual anion-exchange-membrane water electrolysis device (Fig. 8a). The current density of the MEA composed of Ir–H_x_WO_3_/CFP(±) is much higher than that of the MEA composed of benchmark commercial (−)Pt/C + Ir/C(+) under the same cell voltage. At a current density of 10 mA cm^−2^, the cell voltage is 1.78 V for Ir–H_*x*_WO_3_/CFP(±)-based MEA system, which is much less than that of 2.05 V for benchmark commercial (−)Pt/C + Ir/C(+)-based MEA setup (Fig. 8b). Significantly, the Ir–H_*x*_WO_3_/CFP(±) MEA can be stably operated for at least 40 h at a larger current density of 150 mA cm^−2^ (Fig. 8c), demonstrating unprecedented application prospects.

In summary, we have developed a facile electrochemical method to synthesize a highly unique Ir–H_*x*_WO_3_ catalyst for efficient water splitting in challenging neutral media. The intentionally created local acid-like microenvironment around Ir by spontaneous insertion of protons into WO_3_ was profoundly verified by the electrochromic process and corresponding physicochemical characterizations. Operando Raman measurements, selective poisoning and kinetic isotope effect experiments confirm the coherent synergism between Ir and lattice-hydrogen species of H_*x*_WO_3_, that is, the Volmer process is drastically boosted at Ir site to form Ir–H*, followed by spontaneous recombination of Ir–H* and neighboring revitalized WO–H* species to form H_2_ molecular via interfacial Tafel step, as verified by theoretical calculations, thereby keeping the reaction at a high rate. Consequently, the Ir–H_*x*_WO_3_ catalyst breaks the traditional pH-dependent kinetics limitations compared with conventional Ir/C and Pt/C systems, showing a low overpotential of 20 mV at 10 mA cm^−2^ and Tafel slope of 28 mV dec^−1^ in neutral media, closing to those in acidic media. A neutral water electrolysis device assembled with Ir–H_*x*_WO_3_(±) realizes a cell voltage of 1.78 V at a current density of 10 mA cm^−2^ and a high durability of 40 h at a larger current density of 150 mA cm^−2^. Therefore, our study provides insight into tailoring the local reaction environment to design high-performance catalysts in a more rational and precise way.

## Methods

### Chemicals

Iridium(III) chloride hydrate (IrCl_3_·xH_2_O, 298.58), Ammonium metatungstate ((NH_4_)_6_H_2_W_12_O_40_, 2974.32), Ruodium(III) chloride hydrate (RuCl_3_·*x*H_2_O, 207.43), Palladium(II) chloride (PdCl_2,_ 177.32), Chloroplatinic acid hexahydrate (H_2_PtCl_6_·6H_2_O, 517.91), Nickel nitrate hexahydrate (Ni(NO_3_)_2_·6H_2_O, 290.78), Cobalt nitrate hexahydrate (Co(NO_3_)_2_·6H_2_O, 291.03) and Citric acid (C_6_H_8_O_7_, 192.13) were purchased from Aladdin Inc. Commercial 20 wt% Pt/C, 10 wt% Ir/C, 5 wt% Ru/C and 5 wt% Pd/C were purchased from Sigma-Aldrich. The commercial carbon fiber paper (CFP) was purchased from Shanghai Hesen Electric Co.

### Synthesis of WO_3_

Typically, the WO_3_ grown on CFP was papered by traditional hydrothermal processes followed by heating treatment. The details are as follows: 0.89 g ammonium metatungstate and 0.18 g citric acid were dissolved in 35 mL deionized water and stirred to form a clear solution. A piece of carbon fiber paper (CFP, approximately 2 cm × 4 cm × 0.2 mm) was carefully cleaned with concentrated HNO_3_ solution in an ultrasound bath for several minutes. Then, the CFP was cleaned successively by deionized water, acetone, and absolute ethanol. After the cleaning, the CFP was dried at 60 °C for 30 min. Then, it and the aqueous reagent solution were placed together in a 50 mL Teflon-lined stainless-steel autoclave, which was sealed and maintained at 180 °C for 12 h. The as-synthesized material was then taken out, ultrasonically cleaned for 2 min in deionized water, and dried under vacuum at 70 °C overnight. Finally, the collected sample was placed in muffle furnace and heated to 500 °C at a heating rate of 5 °C min^−1^ and held for 4 h to obtain WO_3_ sample.

### Synthesis of Ir–H_*x*_WO_3_

Ir–H_*x*_WO_3_ was synthesized by a potential-cycling method, which was performed using a CHI 760E electrochemical workstation (Shanghai CHI Instruments Company) and a standard three-electrode cell. 1 M PBS (50 mL) contained 0.2 g L^−1^ iridium chloride was taken as electrolyte solution. WO_3_ precursor, a graphite rod and a saturated calomel electrode (SCE) were used as the working electrode (WE), counter electrode (CE) and reference electrode (RE), respectively. On the WE and CE, different potential cycles (100, 200, 300, 400, 500, and 1000) can be carried out between −0.35 and 0 V vs RHE at a scan rate of 100 mV s^−1^. We have found that 500 cycles can convert the precursor sample to Ir–H_*x*_WO_3_.

### Synthesis of *M*–H_*x*_WO_3_ (*M* = Pt, Ru, Pd, Ni, and Co)

*M*–*H*_*x*_WO_3_ (*M* = Ru, Pt, and Pd) were synthesized by the same approach as the preparation of Ir–H_*x*_WO_3_, except that metal precursor was changed to ruodium chloride, chloroplatinic acid or palladiump chloride, respectively. Commercial 20 wt% Pt/C, 5 wt% Ru/C and 5 wt% Pd/C were used as comparison samples. *M–*H_x_WO_3_ (*M* = Ni and Co) were prepared as following steps. 400 μL 20 g L^−1^ nickel nitrate or cobalt nitrate was dropped onto 1 × 1 WO_3_/CFP, and then dried up. The resulting material was reduced in tube furnace at 500 °C for 3 h in H_2_ atmosphere (flow rate: 50 sccm). The same approach was conducted on pure CFP to obtain Ni and Co comparison samples.

### Synthesis of H_*x*_WO_3_

H_*x*_WO_3_ was synthesized by the same approach as the preparation of Ir–H_*x*_WO_3_, except that the pure 1.0 M PBS was taken as electrolyte solution.

### Synthesis of Ir–D_*x*_WO_3_

Ir–D_*x*_WO_3_ was synthesized by the same approach as the preparation of Ir–H_*x*_WO_3_, except that the 1.0 M PBS (D_2_O) contained 0.2 g L^−1^ iridium chloride was taken as electrolyte solution.

### Characterization

Powder X-ray diffraction (XRD) was performed on a Bruker D8 Advance diffractometer (Cu Kα, λ = 1.5418 Å) at the operating voltage of at 40 kV and current of 40 mA. TEM and SEM images were taken on a Hitachi-7700 microscope and Hitachi S-4800 microscope, respectively. A JEOL JEM-2100F with a 200 kV acceleration voltage was used to characterize the HRTEM and HAADF-STEM. Electron paramagnetic resonance (EPR) measurements were performed on a Bruker A300 EPR spectrometer. The UV–Vis diffuse reflectance spectra of the samples were obtained by the UV-3600 spectrophotometer in the region of 200–800 nm, with the BaSO_4_ powder as background. ^1^H solid-state nuclear magnetic resonance (NMR) measurements were performed at room temperature on a Bruker 400 MHz NMR spectrometer. The content of noble metals in the samples was determined by a Thermo IRIS Intrepid II inductively coupled plasma-optical emission spectrometry (ICP-OES). X-ray photoemission spectra (XPS) were obtained on an Escalab 250XI spectrometer with a monochromatized Al Kα source (1486.6 eV) and a base pressure in the lower 2 × 10^−7^ mbar range, and XPS spectra were calibrated with the C 1*s* peak at 284.8 eV. High-resolution XPS spectra were acquired with an analyzer pass energy of 40 eV. The XPS spectra were fitted after subtraction of a Shirley background with the available XPSPEAK 4.1 software.

### Temperature-programmed desorption-mass spectroscopy (TPD-MS) measurement

In the experiment to investigate the effect of Ir on hydrogen species in H_*x*_WO_3_ support, 100 mg of catalyst sample with grain sizes of 60–80 mesh was loaded in a quartz reactor and pretreated with a ultra-pure Ar gas (with a flow rate of 30 ml min^-1^) at 200 °C for 2 h to remove the surface-adsorbed species. The pretreated sample was then cooled to room temperature, following which the sample was heated to 800 °C under the Ar gas flow (30 ml min^−1^) at a heating rate of 2 °C min^−1^.

In the experiments to investigate hydrogen desorption sites, after Ar pretreatment, H_2_ adsorption was carried in H_2_ gas flow (30 ml min^−^) for 1 h, followed by purging with Ar gas (30 ml min^−1^) for 1 h to remove the physically adsorbed H_2_ on the sample surface. Finally, a programmed temperature desorption test under Ar gas flow was performed.

The identification of species desorbed during the thermal process was done by re-analyzing the total ion spectra for the specific mass. The main desorbed specie detected is H_2_ with *m/e* = 2.

### Electrochemical measurements

All electrochemical measurements were performed on a CHI 760E electrochemical workstation (Shanghai CHI Instruments Company) at room temperature. A graphite rod and a saturated calomel electrode were used as the counter electrode and the reference electrode, respectively. The calibration procedure for the reference electrode (SCE) was presented in Supplementary Fig. 32. Ir–H_*x*_WO_3_ Samples were cut into 1 × 1 cm^2^ and directly used as the working electrode. For commercial Pt/C, Ir/C, Ru/C and Pd/C electrodes, 10.0 mg catalyst powder and 25 μl Nafion solution (5 wt%) were dispersed in 1.0 ml ethanol solution by sonication for 30 min to get a homogeneous ink. Then, certain amounts of the ink were loaded on a CFP (1 × 1 cm^2^) and dried under infrared lamp. In Fig. 4, Pt or Ir metal loading was 0.1 mg_metal_ cm^−2^ to obtain appreciable catalytic activity. In the activity evaluation of *M*–H_*x*_WO_3_ systems, for parallel comparison, the loading amount of Ru/C, Pt/C and Pd/C comparison samples on CFP was 58 μg_Ru_ cm^−2^, 50 μg_Pt_ cm^−2^ and 65 μg_Pd_ cm^−2^, respectively. All potentials measured were calibrated vs RHE using the following equation: *E*(RHE) = *E*(SCE) + 0.244 V + 0.0592 × pH. For each HER experiment, cathodic linear sweep voltammetry (LSV) was performed in high-purity H_2_-saturated 1.0 M PBS (pH = 7.03 ± 0.08) or 0.5 M H_2_SO_4_ (pH = 0.35 ± 0.04) at a scan rate of 2 mV s^−1^ (the pH value was determined by pH meter, see Supplementary Fig. 33). All the polarization curves were the steady ones after several scans with iR compensation. The iR compensation was adopted by on-the-fly correction with positive feedback mode, where the points were automatically corrected by instruments with built-in iR compensation. A factor of 95% was applied to measure the resistance value of electrolyte at the open circuit potential. Solution resistances in 1.0 M PBS were 0.4 ± 0.1, 0.6 ± 0.2, 0.8 ± 0.1, 1.1 ± 0.2, and 0.5 ± 0.2 ohm for commercial Pt/C, Ir/C, Ru/C, Pd/C and *M*–H_*x*_WO_3_ samples (*M* = Ir, Pt, Ru, Pd, Co, Ni), respectively. Solution resistances in 0.5 M H_2_SO_4_ were 0.3 ± 0.1, 0.5 ± 0.2, and 0.3 ± 0.1 ohm for commercial Pt/C, Ir/C and Ir–H_*x*_WO_3_ catalysts, respectively. The current density was calculated against the geometric area (1 cm^2^) of the electrode to obtain the specific activity. Electrochemical impedance spectroscopy (EIS) measurements were performed from 10^−2^ to 10^5 ^Hz with an amplitude of 5 mV at an overpotential of 30 mV in 1.0 M PBS solution. The stability measurements were performed by transient accelerating degradation technique (ADT) protocol and static chronopotentiometry test in 1.0 M PBS solution. ADT tests were conducted as follows. Typically, square-wave voltammetry consisting of 10,000 cycles were conducted between a higher potential of 0.15 V_RHE_ and a lower potential of −0.35 V_RHE_. Each cycle was maintained for 4 s. Chronopotentiometry was measured at 10 and 500 mA cm^−2^ to represent typical static long-term stability tests.

### Measurement of pH on the catalyst surface

According to previous work^44^, the pH values on the catalyst surfaces were measured by the rotating ring-disk electrode (RRDE) technique. The potential of Pt ring electrode (RE, 0.1866 cm^−2^) is sensitive to pH and can be used to monitor the variations in the pH on the disk electrode (DE, 0.2475 cm^2^) surface. A three-electrode cell was constructed of the RRDE, graphite rod and a saturated calomel electrode (SCE) as working, counter, and reference electrodes, respectively. Then, the pH dependence of the open circuit potential (*E*_ocp_) in H_2_-saturated 1.0 M PBS solution was measured with Pt RE (Supplementary Fig. 5). The OCP of the Pt electrode would indicate the equilibrium potential of 2H^+^ + 2e^−^ → H_2_, which varies with pH according to the Nernst equation:

3 $$E({{\mbox{V}}}\,{{\mbox{vs.}}}\,{{\mbox{SHE}}})=\frac{{{{-}}}2{{\mbox{.}}}303{RT}}{F}{{\mbox{pH}}}$$

The fugacity of H_2_ is assumed to be equal to unity and *R*, *T,* and *F* are the gas constant, the absolute temperature, and the Faraday constant.

For measuring the pH on the electrode surface, the investigated catalyst was loaded onto the disk electrode. The catalyst ink was prepared by ultrasonically dispersing catalyst powder (5 mg) in 5 wt% Nafion solution (20 μL) and ethanol (480 μL) mixed solution. 10 μL of catalyst ink (10.0 mg mL^−1^) was then transferred onto the disk electrode (catalyst loading: 0.4 mg cm^−2^). The pH measurements on the catalyst surfaces were conducted in 1.0 M PBS solution with the working electrode rotating at a speed of 1600 rpm. Constant potential method was performed on the disk electrode (*E* = 0.1, 0, −0.1, −0.2, −0.3, −0.4, −0.5, −0.6, and −0.7 V_RHE_ for 200 s) to obtain a steady-state current response ( *j*), and OCP was simultaneously measured on the Pt ring electrode. The pH value of the catalyst-loaded DE can be deducted from the pH value of the Pt RE by the following equation:

4 $${c}_{{{{{{{\rm{rt}}}}}},{{{{{\rm{H}}}}}}}^{+}}-{c}_{{{{{{{\rm{rt}}}}}},{{{{{\rm{OH}}}}}}}^{-}}={N}_{{{{{{\rm{D}}}}}}}({c}_{{{{{{{\rm{d}}}}}},{{{{{\rm{H}}}}}}}^{+}}-{c}_{{{{{{{\rm{d}}}}}},{{{{{\rm{OH}}}}}}}^{-}})+(1-{N}_{{{{{{\rm{D}}}}}}})({c}_{\infty,{{{{{{\rm{H}}}}}}}^{+}}-{c}_{\infty,{{{{{{\rm{OH}}}}}}}^{-}})$$

where ${c}_{{{\mbox{rt}}},{{{\mbox{H}}}}^{+}}$ and ${c}_{{{\mbox{d}}},{{{\mbox{H}}}}^{+}}$ are the concentrations of H^+^ on the RE and DE, respectively, ${c}_{{{{\mbox{rt}}},{{\mbox{OH}}}}^{-}}$ and ${c}_{{{{\mbox{d}}},{{\mbox{OH}}}}^{-}}$ are the concentrations of OH^−^ on the RE and DE, respectively, ${c}_{\infty {,{{{{{\rm{H}}}}}}}^{+}}$ and ${c}_{\infty {,{{{{{\rm{OH}}}}}}}^{-}}$ are the concentrations of H^+^ and OH^-^ in the bulk electrolyte, respectively, and *N*_D_   =  0.37 is the collection efficiency of the RE.

### Neutral water electrolysis device

For a neutral water electrolysis device system, the bifunctional Ir–H_*x*_WO_3_/CP catalysts (2 × 2 cm^2^) were both for the anodic OER and cathodic HER. As for benchmark commercial (−)Pt/C + Ir/C(+) partners, homogeneous slurries consisting of catalysts, Nafion solution (5.0 wt.%) and ethanol were air-sprayed onto the carbon fiber paper with an iridium black loading of 2.0 mg cm^−2^ for the anode and 1.0 mg cm^−2^ of Pt/C for the cathode. In all, 1.0 M PBS electrolyte was cycled both on the anodic and cathodic sides by a peristaltic pump, and the flow rate is 80 mL min^−1^. Anion-exchange membrane (Fumasep FAA-3-PK-130) was used to isolate the cathode and anode. Polarization curves were collected from 1.0 to 3.5 V at room temperature under ambient pressure. The current density was calculated against the geometric area (4 cm^2^) of the MEA to obtain the specific activity without iR compensation. The stability test was carried out by galvanostatic electrolysis at a constant current density of 150 mA cm^−2^.

### Operando Raman spectroscopy measurement

Operando Raman spectra were recorded on a LabRAMHR Evolution with an Ar^+^ laser of 514 nm excitation under controlled potentials by the electrochemical workstation. The electrolytic cell was homemade by Teflon with thin round quartz glass plate as cover to protect the objective. The Ir–H_*x*_WO_3_ and H_x_WO_3_ were directly used as working electrode. The Ag/AgCl electrode with inner reference electrolyte of 1.0 M KCl and a Pt wire serves as the reference electrode and the counter electrode, respectively. 1.0 M PBS was used as electrolyte and chronoamperometry measurements were conducted at the potential range from 0 to −0.30 V_RHE_ with the interval of 50 mV. The spectrum was obtained from at least 20 points to ensure accuracy of the information about the samples. The applied voltage–time of each point is 100 s, and the Raman test began when the time exceeded 60 s.

### Computational section

The Density Functional Theory (DFT) calculations were performed in the Vienna Ab initio simulation package (VASP) with the Perdew-Burke-Emzerhof (PBE). The projector augmented wave (PAW) functional was selected as the generalized gradient approximation (GGA) to describe the electron-ion interactions. The cut-off energy of 400 eV and Gaussian electron smearing method with *σ* = 0.05 eV were used. A vacuum of 15 Å was adopted along the *z*-axis. And (5 × 5 × 1) and (2 × 2 × 1) Monkhoest-Pack k-point mesh were used for all samples during electronic structure calculation and the structure optimization, respectively. During structure optimization, the geometry optimization was performed when the convergence criterion on forces became smaller than 0.02 eV Å^−1^ and the energy difference was <10^−4 ^eV. To model the Ir–H_*x*_WO_3_ catalyst, the Ir_10_ clusters were supported on 4 × 4 supercells of WO_3_ (002) with surface O atoms saturated with H atoms. The atoms in the bottom two layers of WO_3_ (002) were kept frozen while the remaining were allowed to relax during the slab calculations. Gibbs free energy of X species (*X* = H_2_O and H) adsorption is calculated by

5 $$\triangle G={\triangle E}_{{{\mbox{X}}}/{{\mbox{surf}}}}-{\triangle E}_{{{\mbox{surf}}}}-{{{\mbox{u}}}E}_{{{\mbox{X}}}}+{\triangle E}_{{{\mbox{ZPE}}}}-{{{\mbox{T}}}\triangle S}_{{{\mbox{H}}}}$$

where *E*_X/surf_ is the total energy of the surface with adsorbate, *E*_surf_ is the energy of the clean surface, *E*_X_ is the energy of adsorbate, Δ*E*_ZPE_ represents the zero-point energy of the system, and *T*Δ*S*_H_ is the contribution from entropy.

## Supplementary information

Supplementary information

Peer Review File

Description of Additional Supplementary Files

Supplementary Movie 1

Supplementary Movie 2

Supplementary Movie 3

Supplementary Movie 4

Supplementary Movie 5

## Data Availability

The authors declare that all data supporting the findings of this study can be found in the manuscript and Supplementary Information, or are available from the corresponding authors upon request.
