# Supplementary material for: Tailoring a local acid-like microenvironment for efficient neutral hydrogen evolution
Source: Nat Commun. 2023 Jul 14;14:4209. doi: 10.1038/s41467-023-39963-8 (PMC10349089; doi:10.1038/s41467-023-39963-8)
Supplement: Supplementary file 1 — Supplementary information [file 41467_2023_39963_MOESM1_ESM.pdf]

Supplementary Information

Tailoring a local acid-like microenvironment for efficient neutral hydrogen evolution

Xiaozhong Zheng[1], Xiaoyun Shi[1], Honghui Ning[1], Rui Yang[1], Bing Lu[1], Qian Luo[1], Shanjun Mao[1], Lingling Xi[1], Yong Wang[1,2]*

[1]*Advanced Materials and Catalysis Group, Center of Chemistry for Frontier Technologies, State Key Laboratory of Clean Energy Utilization, Institute of Catalysis, Department of Chemistry, Zhejiang University, Hangzhou 310028, P. R. China.*

[2]*College of Chemistry and Molecular Engineering, Zhengzhou University, Zhengzhou 450001, China*

**Corresponding author E-mail: chemwy@zju.edu.cn*

This file includes:

Supplementary Figures 1 to 33

Supplementary Tables 1 to 3

Supplementary References

Table of Contents

1. Supplementary Figures..S3

2. Supplementary Tables..S36

3. Supplementary References S40

1. Supplementary Figures

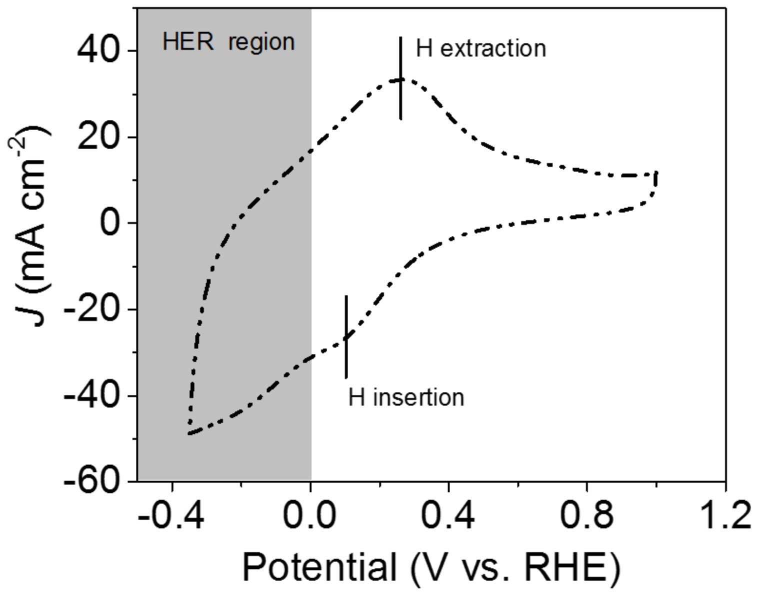

Supplementary Figure 1. Redox properties of WO_3. CV curve of the WO_3 from -0.35 ~ 1.0 V_{RHE} at 50 mV s^{-1}. In HER potential region, WO_3 experienced hydrogen insertion process.

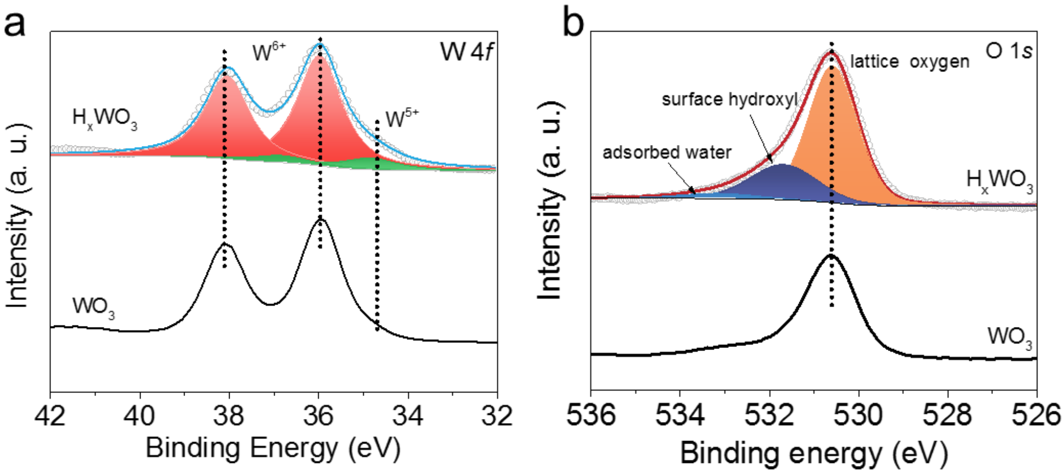

Supplementary Figure 2. XPS spectra of WO_3 and H_xWO_3. (a) High-resolution spectra for the W 4*f* of WO_3 and H_xWO_3. (b) High-resolution spectra for the O 1*s* of of WO_3 and H_xWO_3. These results confirm the formation of W^{5+}-OH after hydrogen insertion in H_xWO_3.

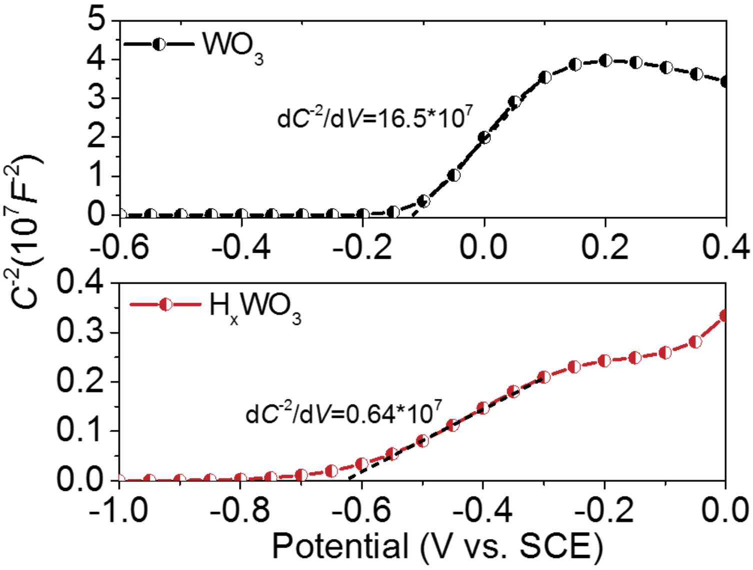

Supplementary Figure 3. Analysis of electrode surface properties. Mott-Schottky (M-S) plots of WO_3 and H_xWO_3.

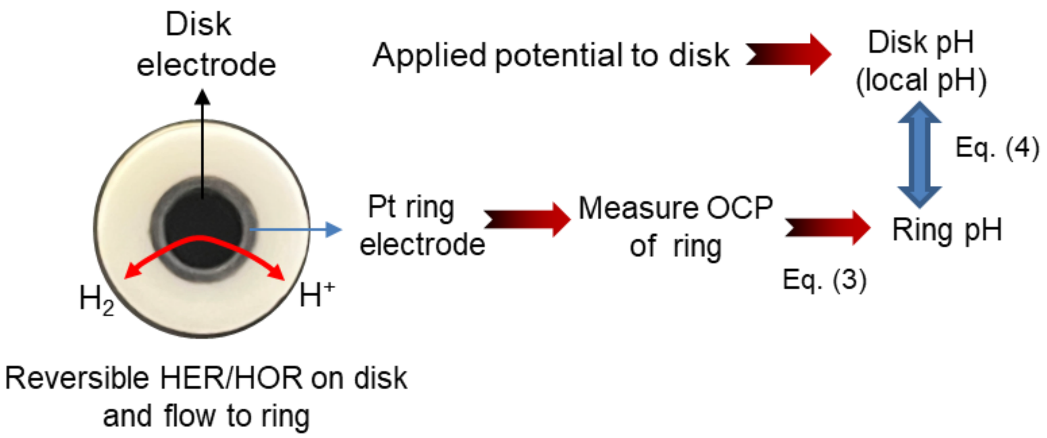

Supplementary Figure 4. Principles for detecting local pH. Schematic diagram for monitoring pH on the electrode surface using an RRDE technology, the equations (3) and (4) are given in Method section.

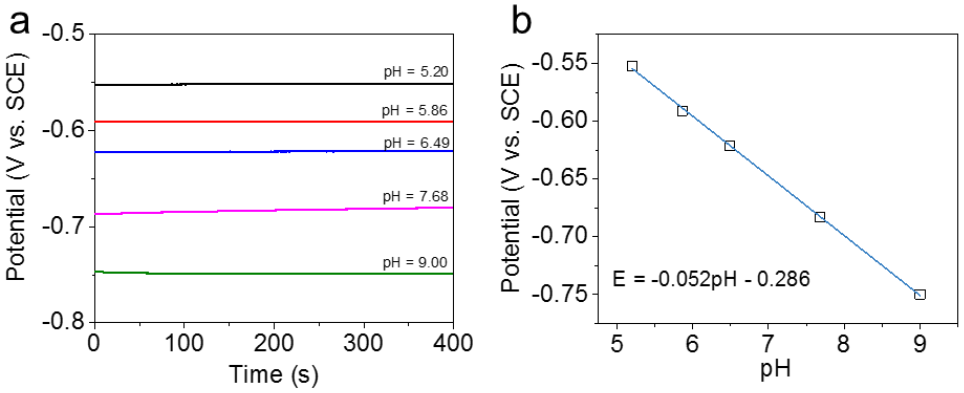

Supplementary Figure 5. The relationship between E_{ocp} of Pt ring electrode and pH. (a) Time and (b) pH dependence of open circuit potential (E_{ocp}) for Pt ring electrode. The measurement was performed in 1.0 M PBS solutions, and the pH of the PBS solutions was changed by adding H_2SO_4 or KOH.

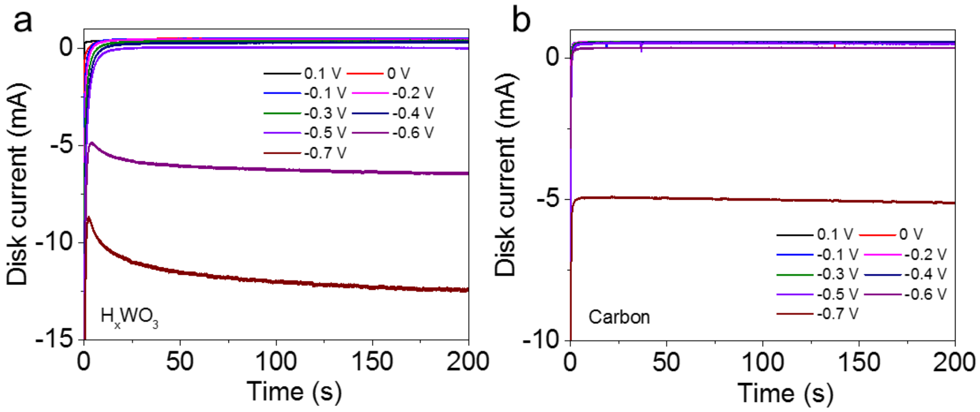

Supplementary Figure 6. The steady-state current of electrodes at different potentials. J-T curves of H_xWO_3 (a) and carbon (b) support in different potentials.

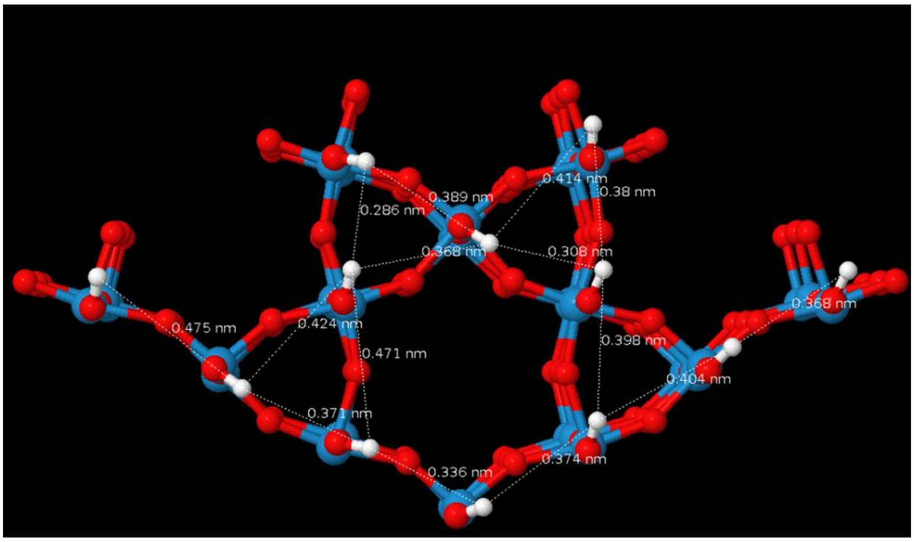

Supplementary Figure 7. Structural analysis of H_xWO_3 model. The distance of hydride species on H_xWO_3.

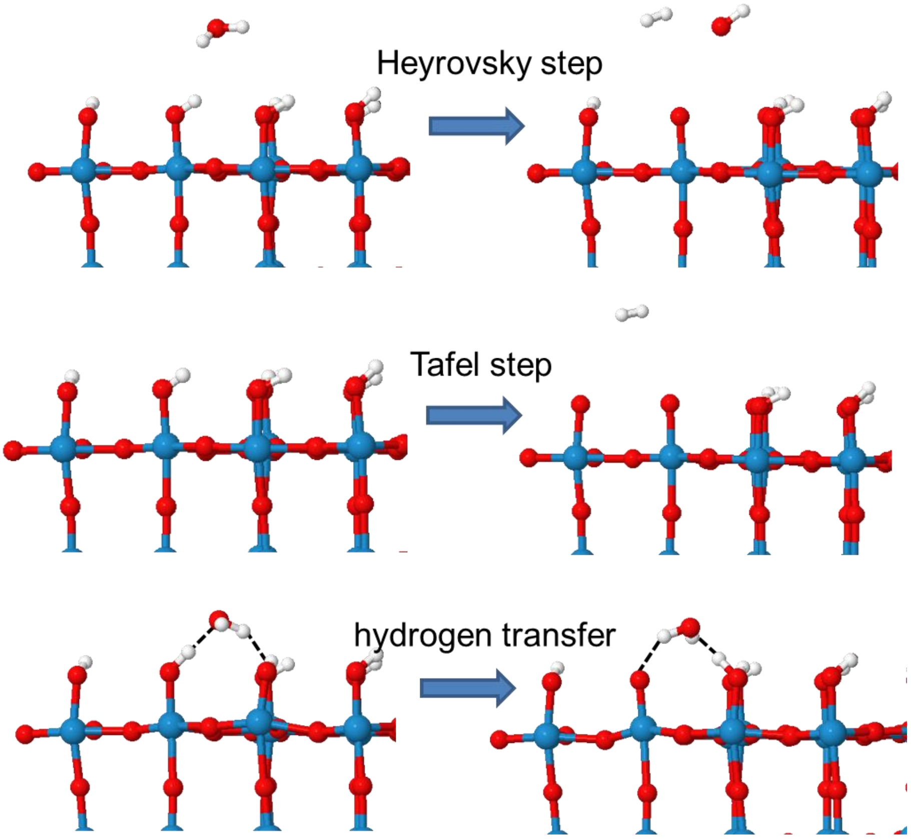

Supplementary Figure 8. Exploration of HER mechanism on H_xWO_3 model. HER Configurations of H_xWO_3 involved in H-H coupling (Heyrosky and Tafel steps) and H-H transfer process.

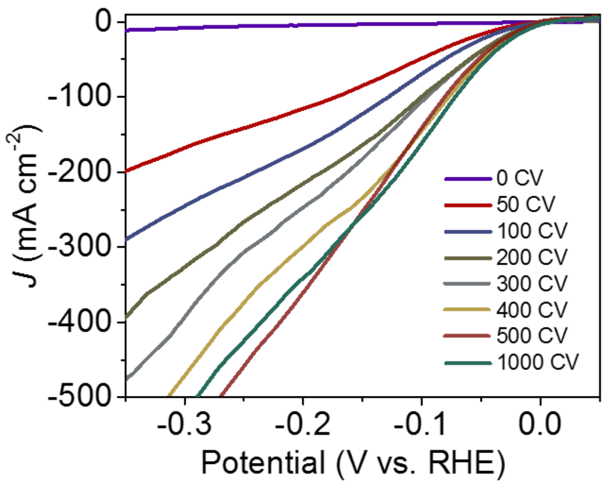

Supplementary Figure 9. Activation process of Ir-H_xWO_3. HER polarization curves of the Ir-H_xWO_3 with 95% iR compensation after different numbers of potential cycles were performed on it.

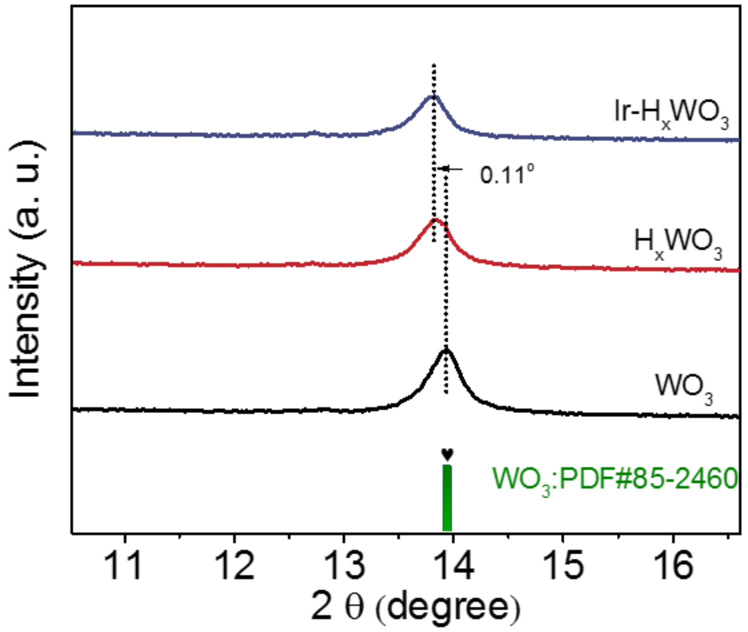

Supplementary Figure 10. Analysis of XRD data. The XRD patterns of WO_3, H_xWO_3 and Ir-H_xWO_3 catalysts in the small range.

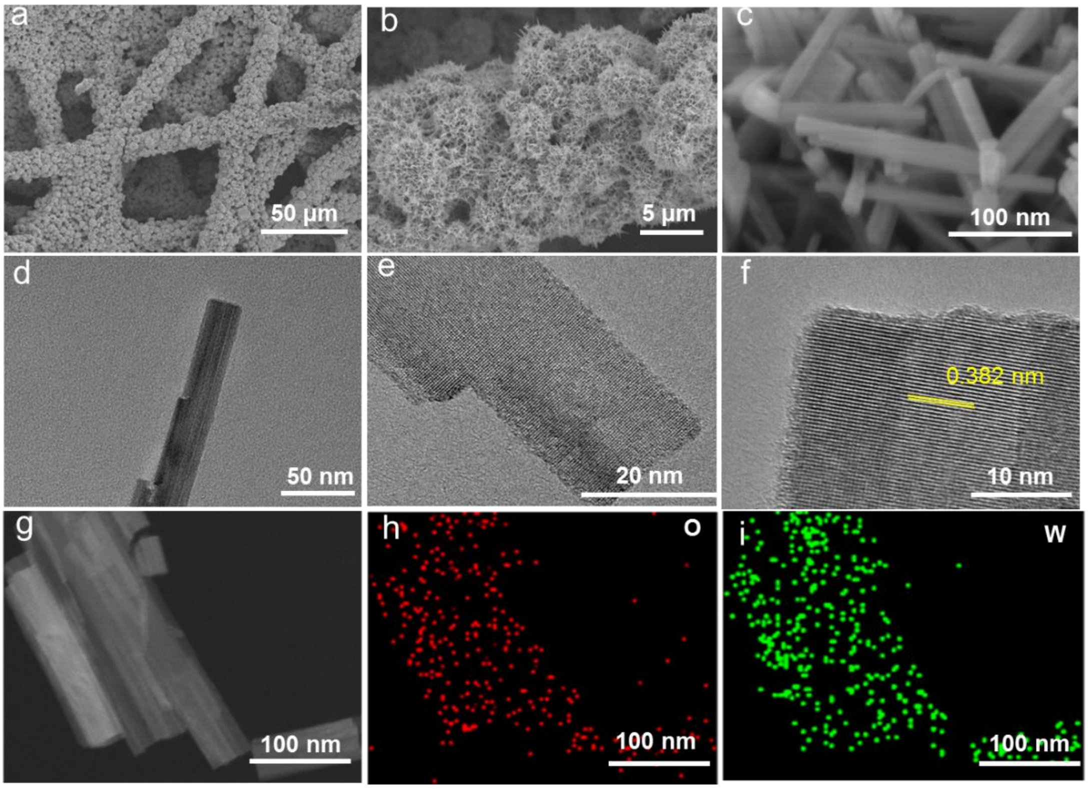

Supplementary Figure 11. **Morphological characterization of WO_3.** (a-c) SEM images of WO_3 nanorods grown on CFP at different resolutions. (d-f) TEM and HRTEM images of WO_3 nanorods. (g-i) HAADF-STEM image and corresponding EDS maps of WO_3 for O and W.

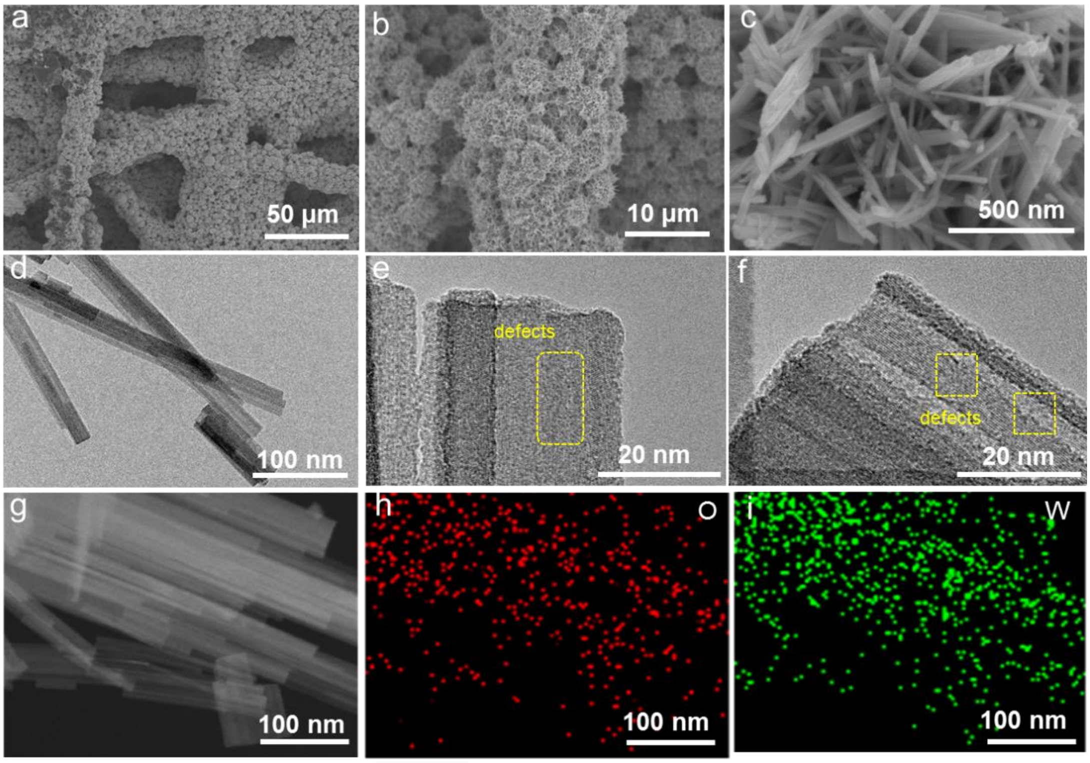

Supplementary Figure 12. **Morphological characterization of H_xWO_3.** (a-c) SEM images of H_xWO_3 nanorods grown on CFP at different resolutions. (d-f) TEM and HRTEM images of H_xWO_3 nanorods. (g-i) HAADF-STEM image and corresponding EDS maps of H_xWO_3 for O and W.

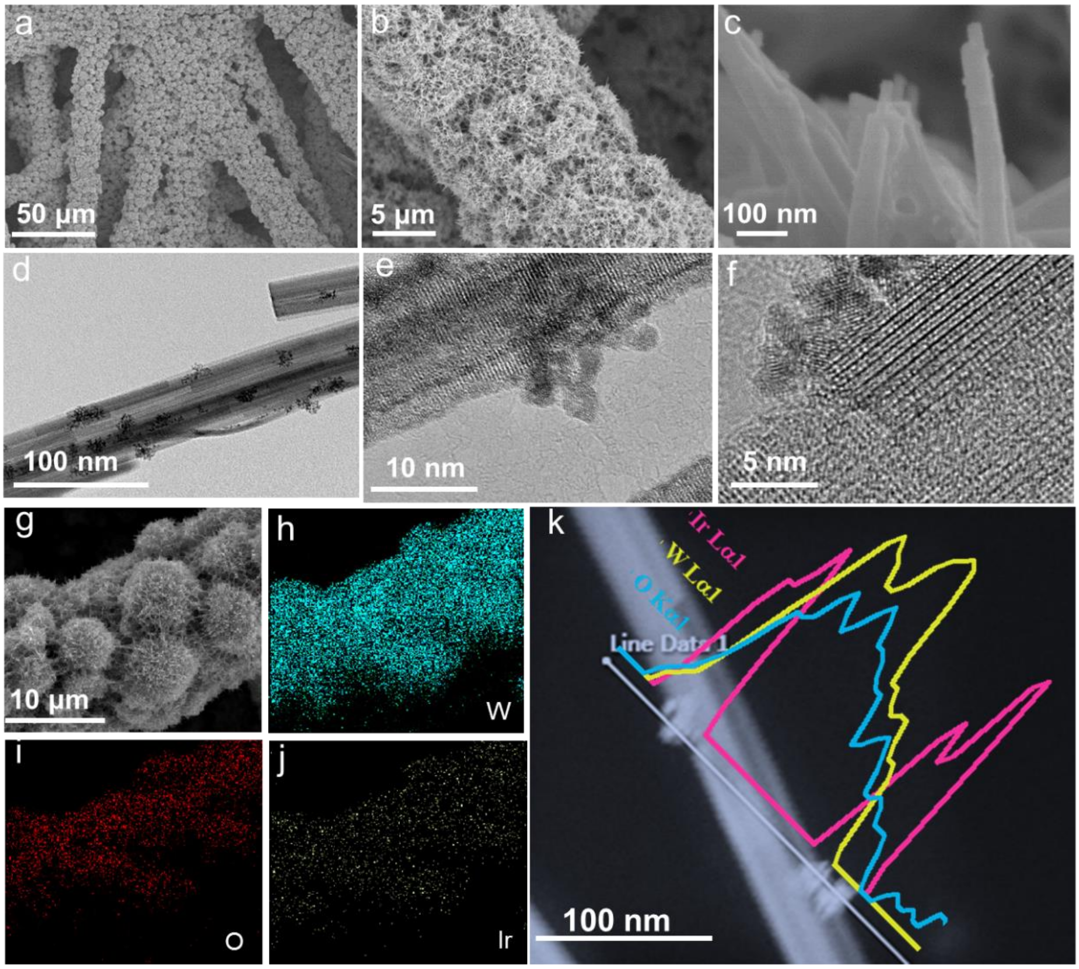

Supplementary Figure 13. Morphological characterization of Ir-H_xWO_3. (a-c) SEM images of Ir-H_xWO_3 nanorods grown on CFP at different resolutions. (d-f) TEM and HRTEM images of H_xWO_3 nanorods; (g-j) The EDS maps of of Ir-H_xWO_3 for Ir, W and O elements. (k) The line-scan profile of Ir-H_xWO_3 for Ir, W and O elements.

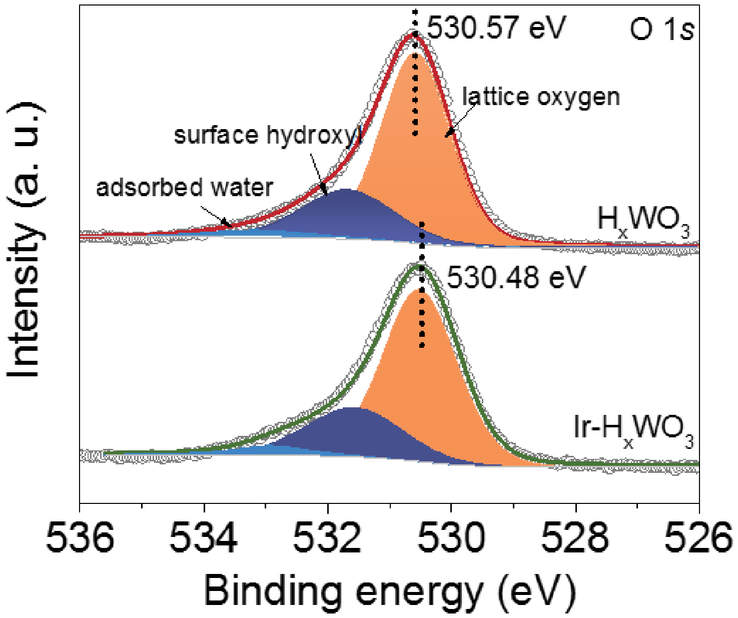

Supplementary Figure 14. Analysis of O 1*s* data. High-resolution O 1*s* spectra of Ir-H_xWO_3 and H_xWO_3.

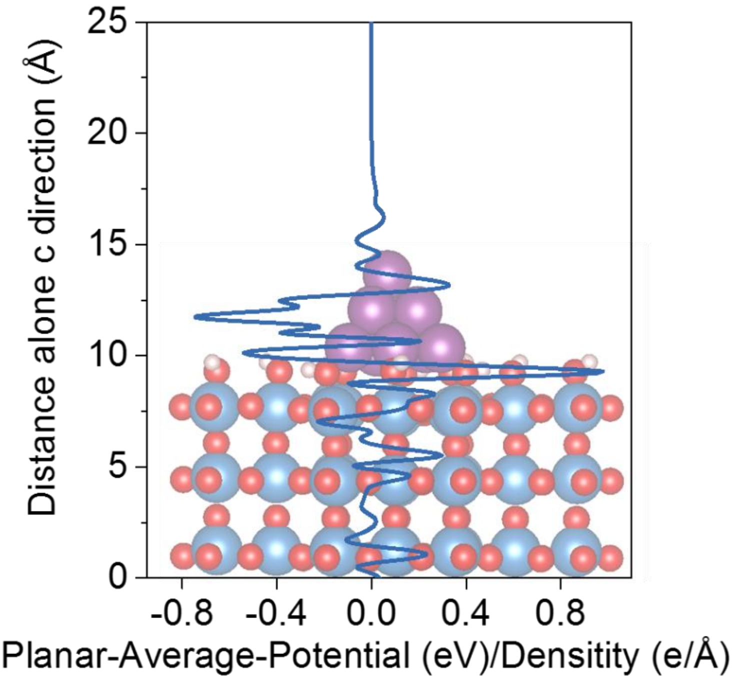

Supplementary Figure 15. Analysis of interfacial built-in electric fields. Planar average potential of Ir_{10}-H_xWO_3 along the Z-direction.

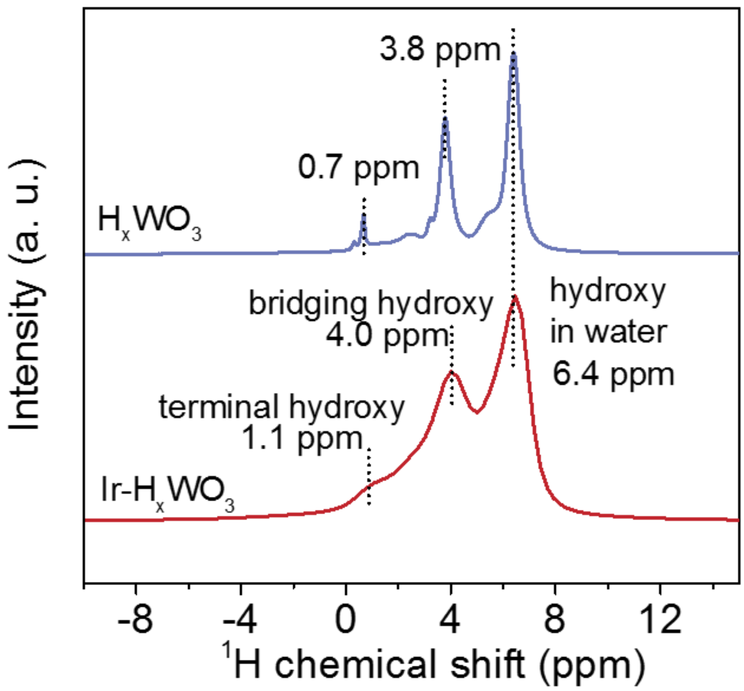

Supplementary Figure 16. Analysis of chemical environment of hydrogen species. ^1H NMR spectra of Ir-H_xWO_3 and H_xWO_3.

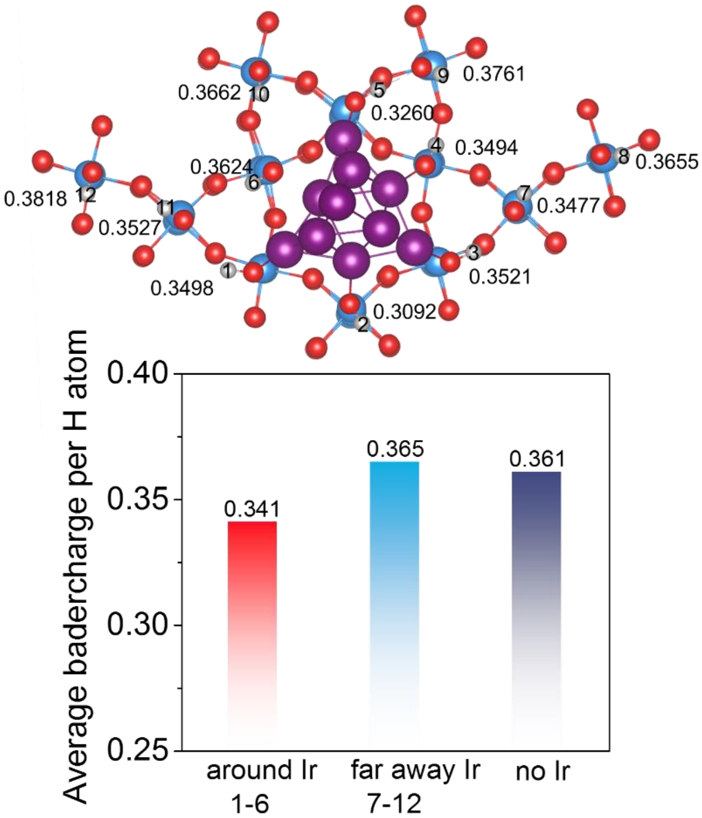

Supplementary Figure 17. **Analysis of Bader charge.** The bader charge of different H atoms (H_1-H_{12}) in Ir-H_xWO_3 and corresponding average bader charges values of H_1-H_6 (around Ir), H_7-H_{12} (far away from Ir) and pure H_xWO_3.

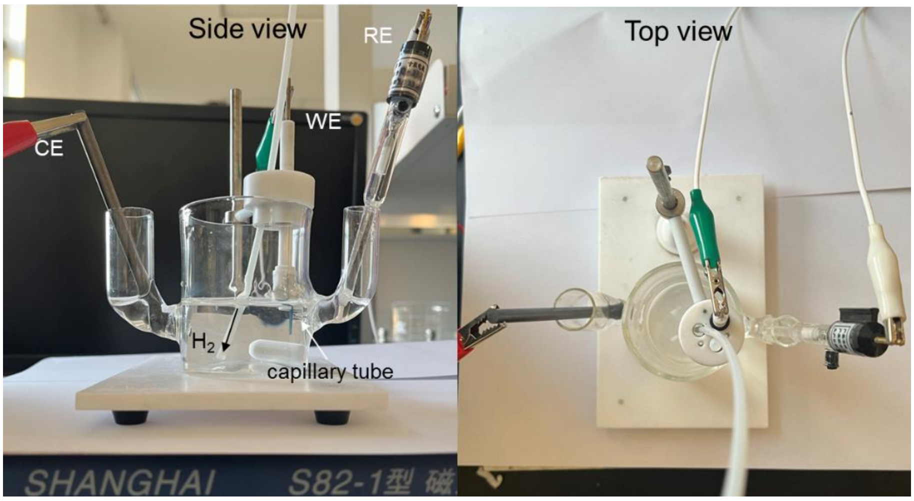

Supplementary Figure 18. **Electrochemical cell set-up.** The side and top views of a conventional three-electrode electrochemical cell.

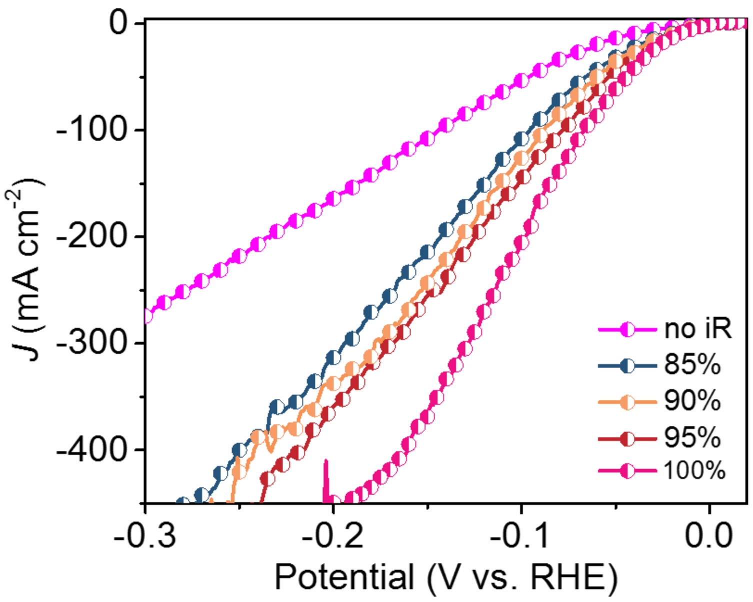

Supplementary Figure 19. Analysis of different levels of iR compensation. The polarization curves of Ir-H_xWO_3 with different levels of iR compensation. The polarization curve with 100% iR compensation is clearly overcorrected. Therefore, the data of 95% iR compensation is presented in Fig. 4a.

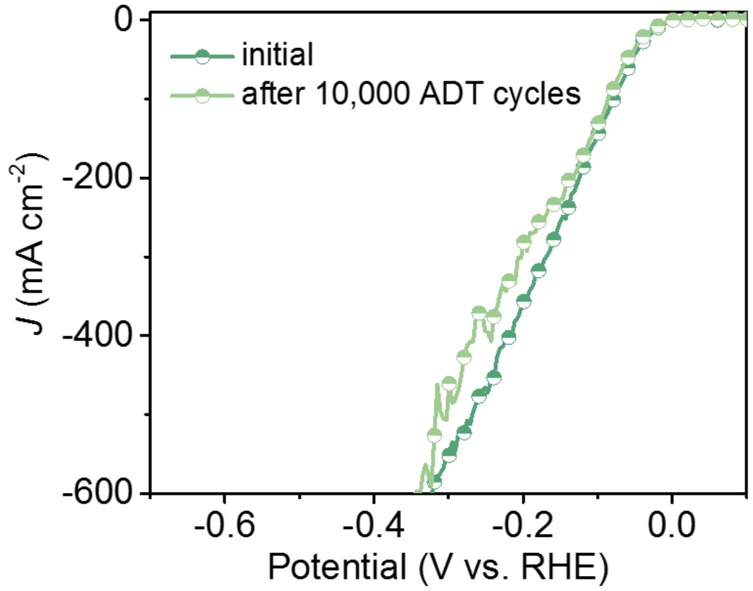

Supplementary Figure 20. The stability test by ADT cycles. HER polarization curves of Ir-H_xWO_3 with 95% iR compensation before and after 10,000 ADT cycles.

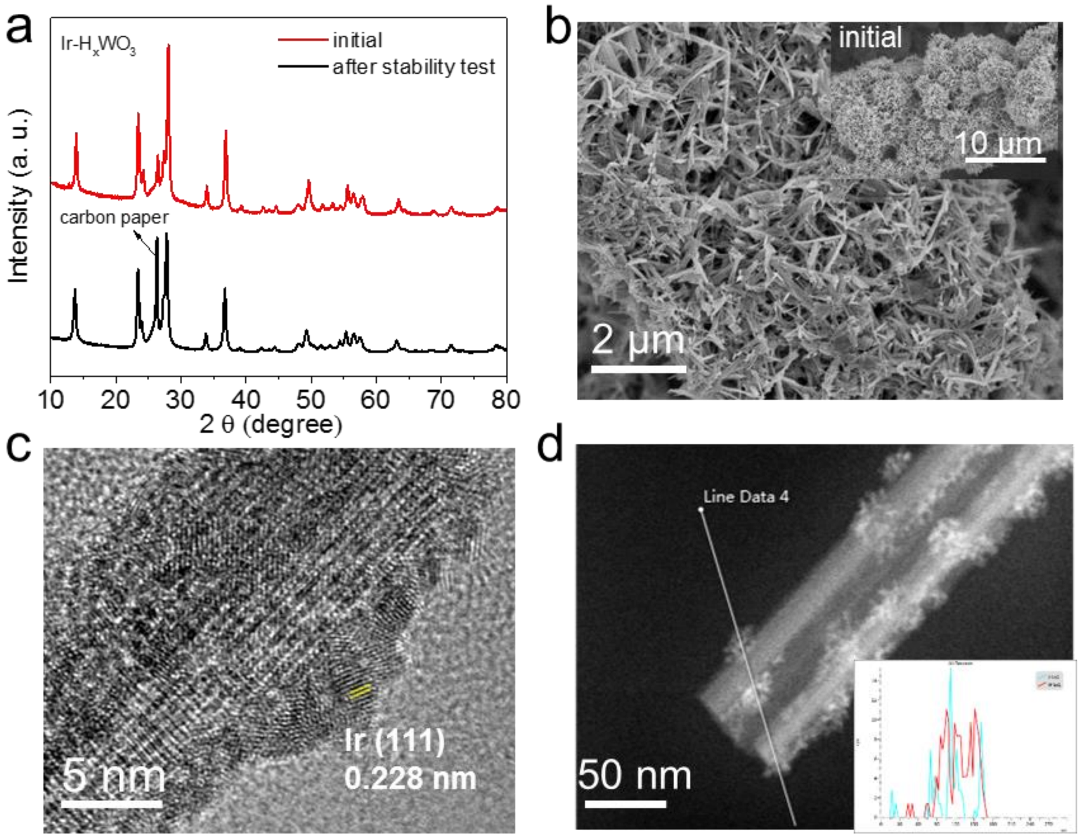

Supplementary Figure 21. Structural analysis of catalysts after stability test. (a) The XRD patterns of Ir-H_xWO_3 before and after stability test. (b,c) The SEM and HRTEM images of Ir-H_xWO_3 after stability test, the inset in (b) giving the initial morphology of catalyst. (d) Line-scan profile of postmortem Ir-H_xWO_3 for Ir, W and O elements.

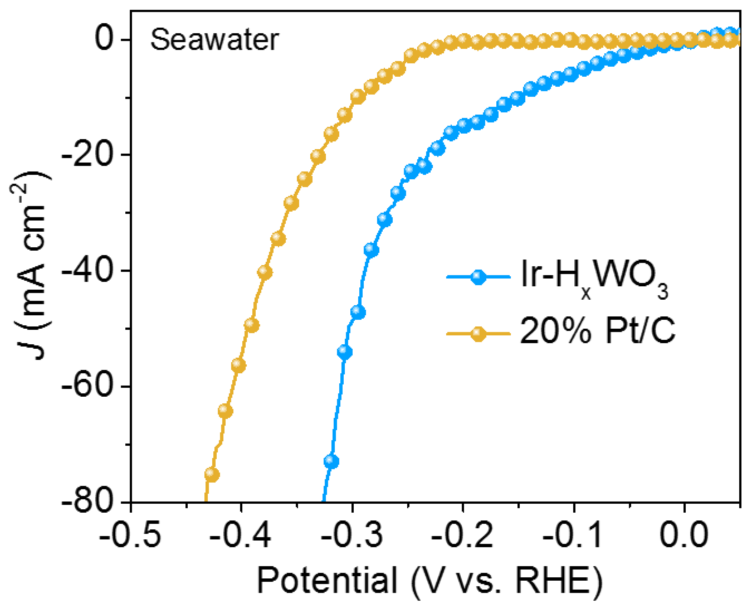

Supplementary Figure 22. The HER performance of catalysts in natural seawater. The LSV plots of Ir-H_xWO_3 and 20% Pt/C in nature seawater with 95% iR compensation.

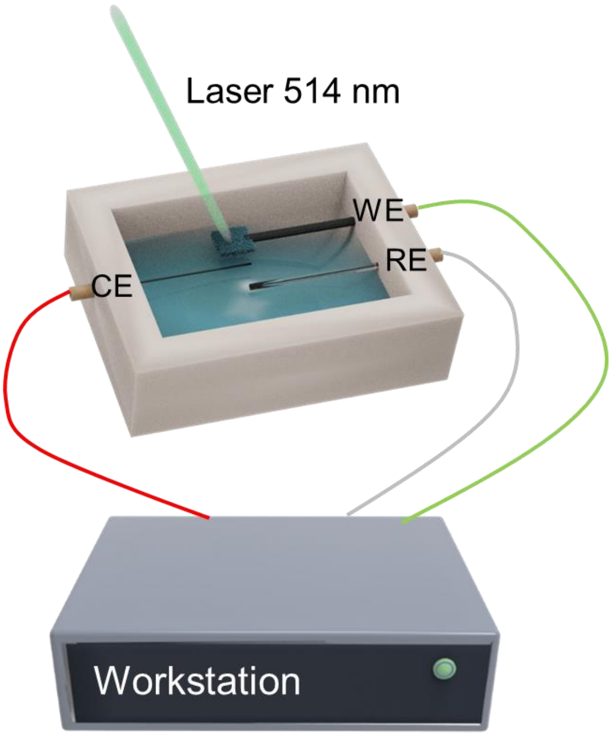

Supplementary Figure 23. **Electrochemical set-up used for operando study in this work.** The illustration of operando Raman spectroscopy setup.

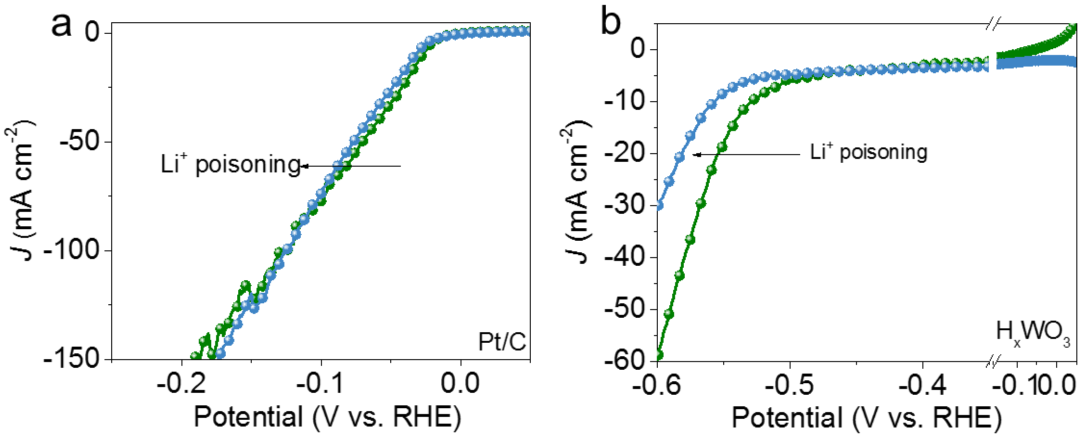

Supplementary Figure 24. Effect of Li^+ poisoning on the catalyst. The LSV plots of 20% Pt/C (a) and H_xWO_3 support (b) with 95% iR compensation before and after Li^+ poisoning treatments.

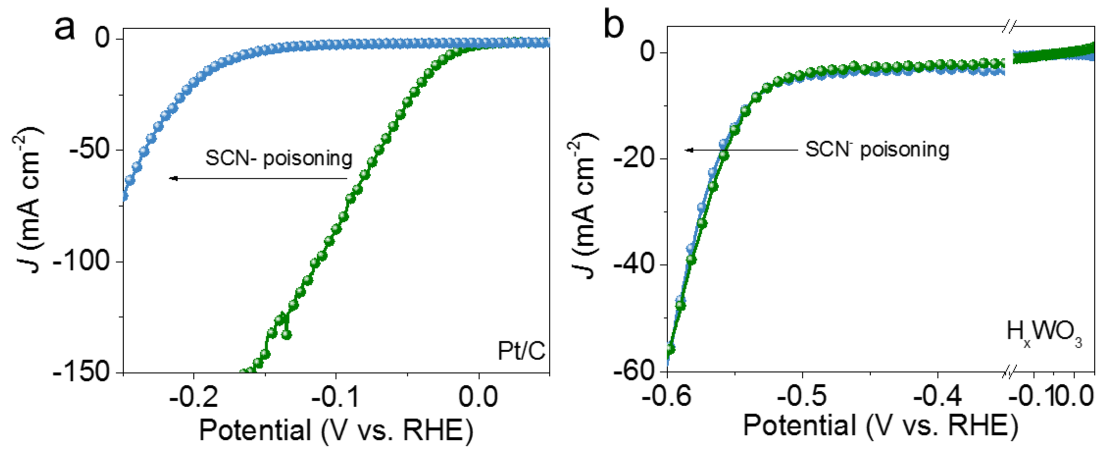

Supplementary Figure 25. Effect of SCN$^-$ poisoning on the catalyst. The LSV plots of 20% Pt/C (a) and H_xWO_3 support (b) with 95% iR compensation before and after SCN- poisoning treatments.

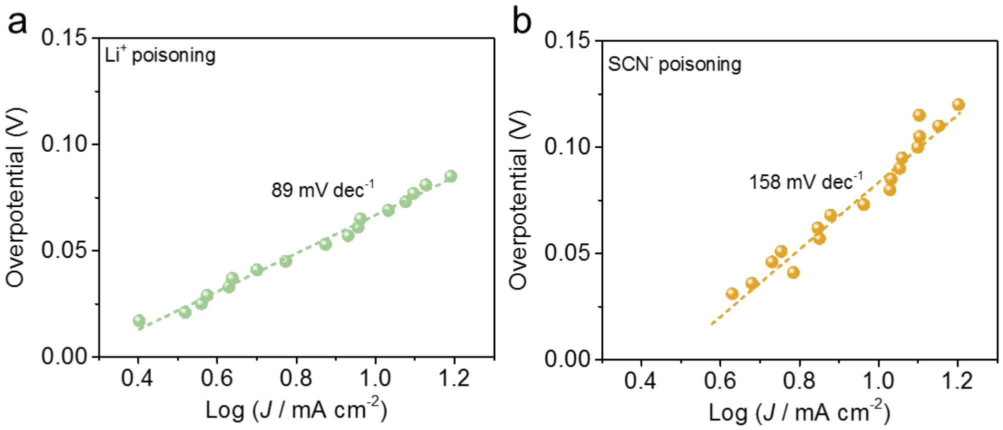

Supplementary Figure 26. Reaction kinetic analysis of catalyst after Li+ and SCN- poisoning. The Tafel plots of Ir-H_xWO_3 after Li^+ poisoning (a) and SCN^- poisoning (b).

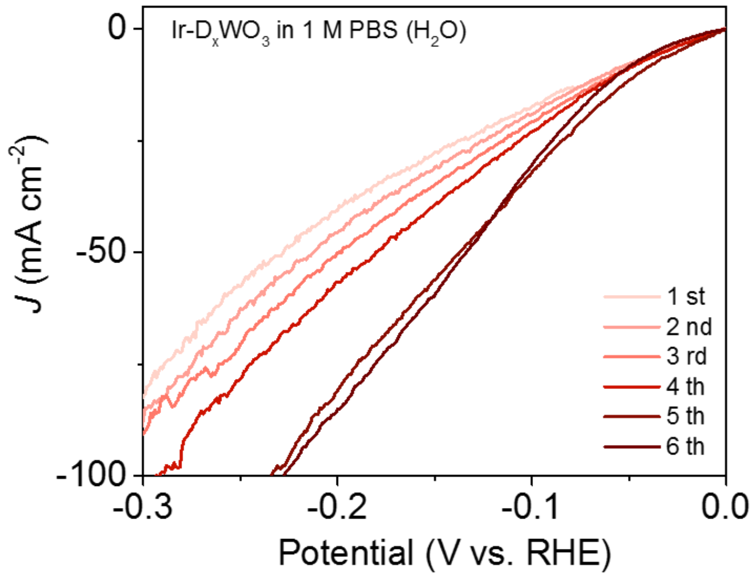

Supplementary Figure 27. The activation process of Ir-D_xWO_3. The LSV plots of Ir-D_xWO_3 in 1.0 M PBS (H_2O) solution with 95% iR compensation after different numbers of HER cycles were performed on it.

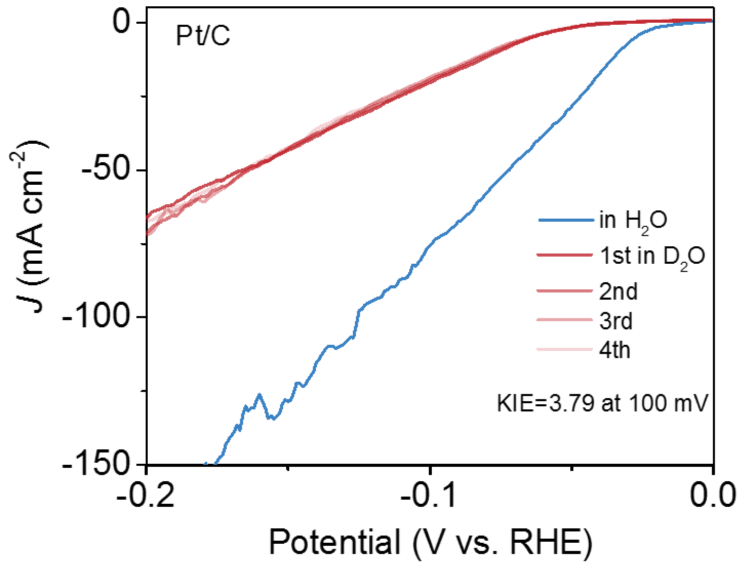

Supplementary Figure 28. KIE effect on Pt/C catalyst. The LSV plots of Pt/C in 1.0 M PBS (D_2O) solution with 95% iR compensation after different numbers of HER cycles were performed on it.

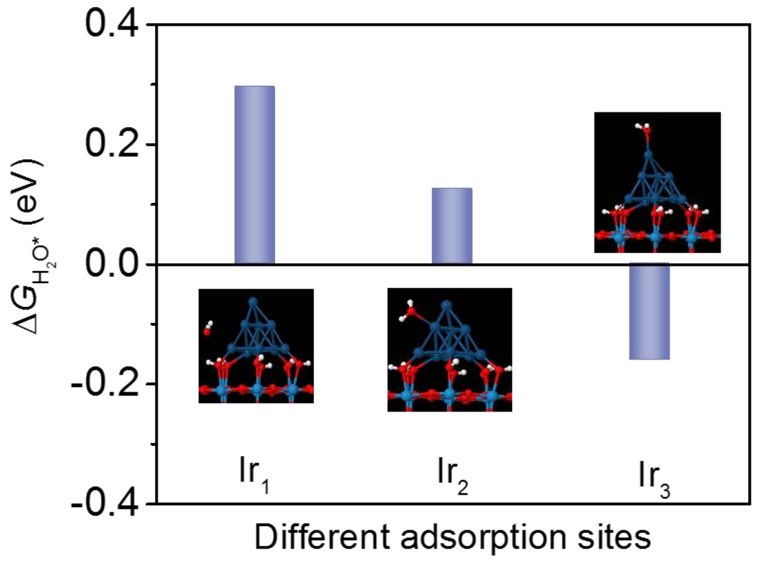

Supplementary Figure 29. Adsorption behavior of water molecules. The absorbed free energy of H_2O at different Ir sites

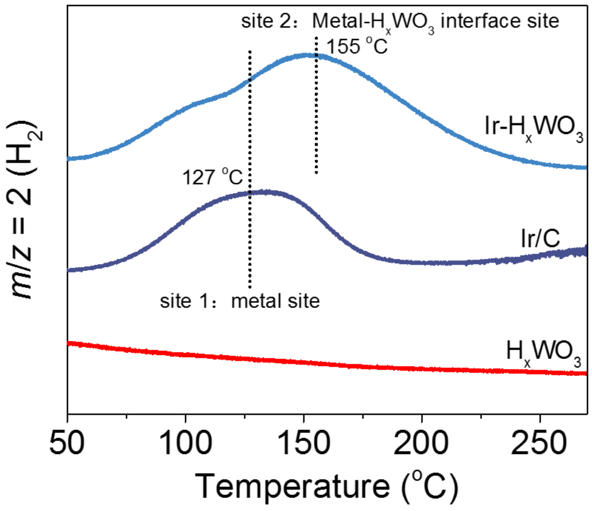

Supplementary Figure 30. Analysis of H_2-TPD data. The H_2-TPD patterns of Ir-H_xWO_3, commercial Ir/C and H_xWO_3 samples.

Supplement: The H_2-TPD profile of commercial Ir/C catalyst exhibits a single desorption peak centered at 127 °C, which is ascribed to the desorption of atomic H over the metallic Ir surface. As we expected in Ir-H_xWO_3 sample, in addition to the observation of H_2 desorption site located on the metal surface, the unique dehydrogenation signal over interface of Ir metal and H_xWO_3 support (155 °C) is also detected, which is consistent with previous results in the literature[1-3]. Moreover, by comparing the amount of dehydrogenation at different sites, we found that H_2 desorption at the interface site is dominant.

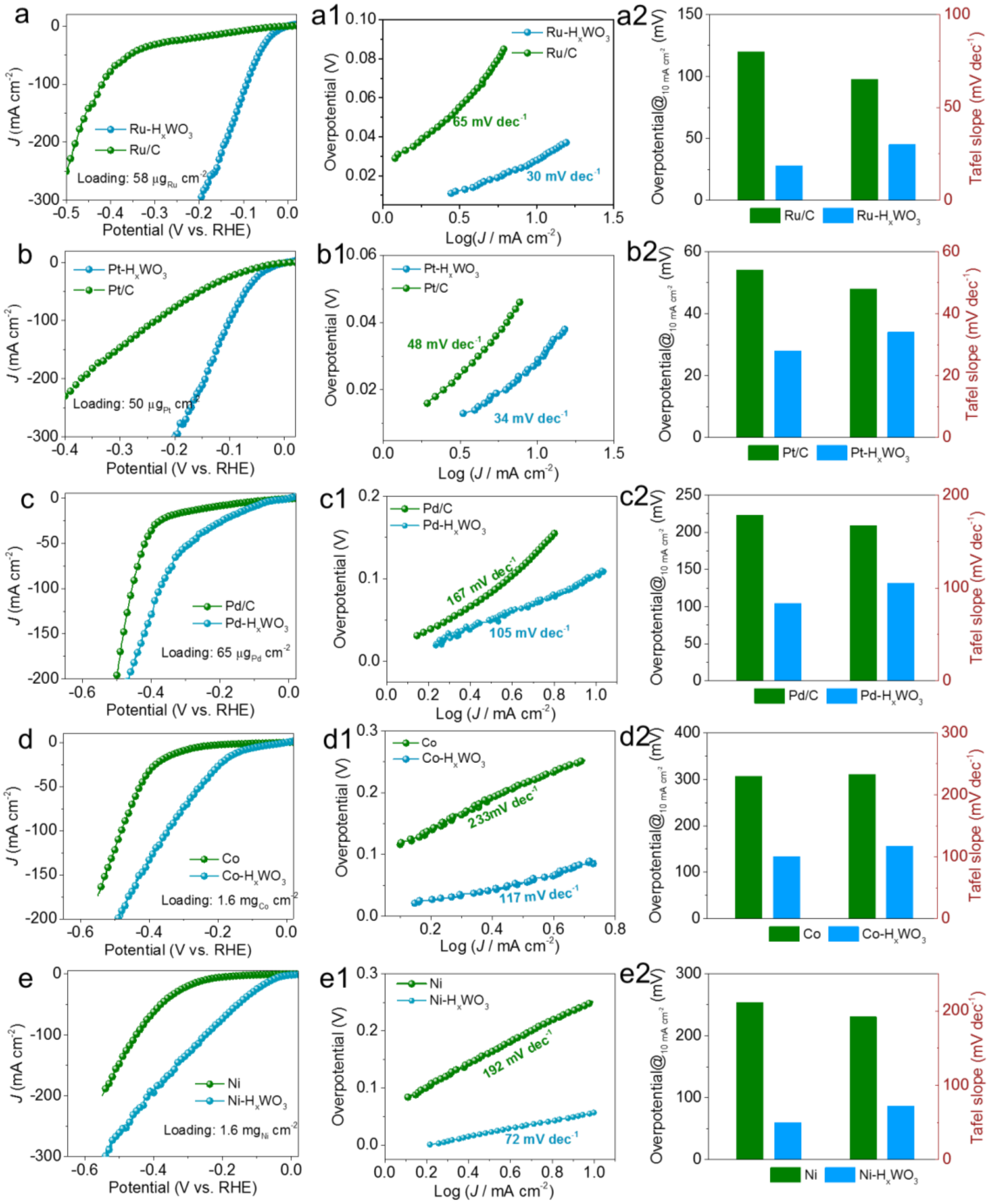

Supplementary Figure 31. **Neutral HER performance of M-H_xWO_3 and M-Carbon systems.** The LSV curves, Tafel plots and corresponding activity comparison of M-H_xWO_3 and M-Carbon systems (M = Ru, Pt, Pd, Co, Ni) in 1.0 M PBS solution. Ru: a-a2; Pt: b-b2; Pd: c-c2; Co: d-d2; Ni: e-e2. All electrochemical data were corrected for 95% iR drop.

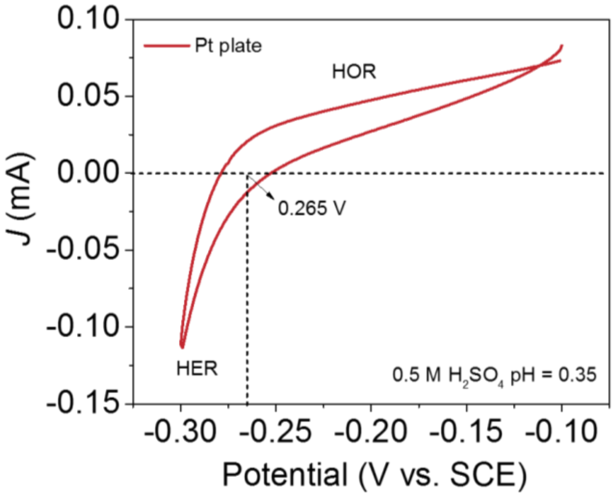

Supplementary Figure 32. **Reference electrode calibration.** CV curve of a Pt plate (area: 1 cm^{-2}) in high-purity hydrogen-saturated 0.5 M H_2SO_4 solution (pH = 0.35) at a scan rate of 2 mV s^{-1}. The average of the two potentials at which the current crossed zero was regarded as the thermodynamic potential for the hydrogen electrode reaction[4]. The standard potential (vs. RHE) of the saturated calomel electrode is calibrated to be approximately 0.244 V.

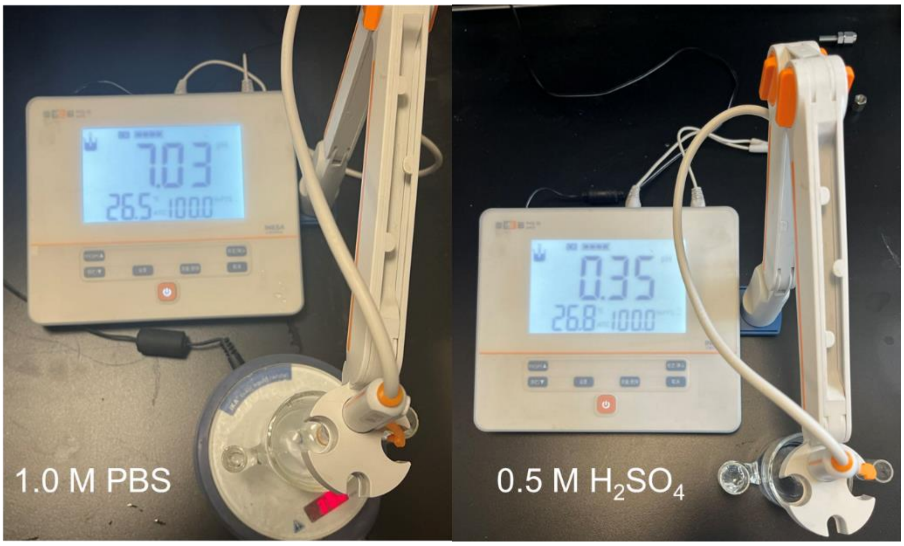

Supplementary Figure 33. **pH measurements of the electrolyte.** The pH of 1.0 M PBS and 0.5 M H_2SO_4 electrolyte measured by pH meter.

2. Supplementary Tables

Supplementary Table 1. Noble metal content of M-H_xWO_3 (M = Ir, Ru, Pt, Pd), Ir/C, Pt/C, Ru/C and Pd/C determined by inductively coupled plasma optical emission spectrometry (ICP-OES).

Catalysts	Initial	After long-term test
Ir-H_xWO_3/CFP	[a] 2.8%	[a] 2.6%
	[b] 47 μg cm^{-2}	[b] 40 μg cm^{-2}
Ru-H_xWO_3/CFP	58 μg cm^{-2}	/
Pt-H_xWO_3/CFP	50 μg cm^{-2}	/
Pd-H_xWO_3/CFP	65 μg cm^{-2}	/
Commercial 10 wt% Ir/C	9.8 %	/
Commercial 20 wt% Pt/C	19.6 %	/
Commercial 5 wt% Ru/C	4.9 %	/
Commercial 5 wt% Pd/C	4.9 %	/

[a] Ir loading normalized to H_xWO_3 support, the calculation formula is as follows: Ir wt% = $\frac{m\,(Ir)}{m(Ir\text{-}H_xWO_3/CFP)-m(CFP)}$ * 100 %.

[b] Ir loading normalized to geometric area of CFP support, the calculation formula is as follows: Ir wt% = $\frac{m\,(Ir)}{A(CFP)}$.

Supplementary Table 2. Comparison of HER activities with various recently reported state-of-the-art catalysts in neutral electrolyte.

**Catalysts**	**η_{10} /mV**	**Tafel /mV dec^{-1}**	**Electrolyte**	***iR* compensation**	**Stability**	**References**
**Ir-H_xWO_3**	20	28	1 M PBS	95%	100 h @ 10 mA cm^{-2}<br>40 h @ 500 mA cm^{-2}<br>10,000 ADT cycles	This work
**$Ni_{0.1}Co_{0.9}P$**	125	109	1 M PBS	100%	20 h @ 30 mA cm^{-2}	*Angew. Chem. Int. Ed.,* 2018, 130, 15671
**CrO_x/Ni-Cu**	48	64	1 M PBS	100%	24 h @ -0.1 V_{RHE} ~ 33 mA cm^{-2}	*Nat. Energy,* 2019, 4, 107
**N-Co_2P/CC**	42	64	1 M PBS	100%	3000 CV cycles	*ACS Catal., 2019, 9, 3744*
**Pt_{SA}-NT-NF**	24	30	1 M PBS	90%, *R*~ 3 Ω	24 h @ 10 mA cm^{-2}	*Angew. Chem., Int. Ed.,* 2017, 56, 13694
**Mn-Co-P/Ti**	86	82	1 M PBS	100%	1000 CV cycles<br>10 h @ -0.096 V_{RHE} ~ 10 mA cm^{-2}	*ACS Catal.,* 2017, 7, 98
**$Ni(S_{0.5}Se_{0.5})_2$**	124	81	1 M PBS	-	2000 CV cycles<br>20 h @ -0.125 V_{RHE} ~ 10 mA cm^{-2}	*J. Mater. Chem. A,* 2019, 7, 16793
**$Ni_{0.89}Co_{0.11}Se_2$**	82	78	1 M PBS	-	40 h @ -0.2 V_{RHE} ~ 45 mA cm^{-2}	*Adv. Mater.,* 2017, 29, 1606521
**MoP700**	196	79	1 M PBS	*iR*-free	4000 CV cycles	*ACS Catal.,* 2019, 9, 8712
**N-Ni**	64	108	1 M PBS	100%	18 h @ 20 mA cm^{-2}	*J. Am. Chem. Soc.,* 2017, 139, 12283
**Co-HNP/CC**	85	38	1 M PBS	100%, *R*~1.7-2 Ω cm^2	20 h @ 150 mA cm^{-2}	*Angew. Chem., Int. Ed.,* 2016, 55, 6725
**CoP/Co–MOF**	49	63	1 M PBS	100%	60,000 s @ 20 and 50 mA cm^{-2}	*Angew. Chem., Int. Ed.,* 2019, 58, 4679
**Karst NF**	110	99	-	90%	10 h @ -0.21 V_{RHE} ~ 10 mA cm^{-2}	*Energy Environ. Sci.,* 2020, 13, 174
**$Ni_{0.33}Co_{0.67}S_2$**	72	68	1 M PBS	100%	20 h @ -0.07 V_{RHE} ~ 10 mA cm^{-2}	*Adv. Energy Mater.,* 2015, 5, 1402031

**$Cu_{0.08}Co_{0.92}P$**	81	83.5	0.5 M $KHCO_3$	100%	3000 CV cycles 20 h @ 10 mA cm^{-2}	*Appl. Catal., B,* 2020, 265, 118555
**Pt/np-$Co_{0.85}Se$**	55	35	1 M PBS	100%	40 h @ -0.05 V_{RHE} ~ 10 mA cm^{-2}	*Nat. Commun.* 2019 10, 1743

Supplementary Table 3. Comparison of HER activities with various recently reported state-of-the-art catalysts in nature seawater.

Electrocatalysts	η_{10} / mV	Electrolyte	References
**Ir-H_xWO_3**	150	Natural seawater	This work
**$Co_{0.31}Mo_{1.69}C$@NC**	312	Natural seawater	*Adv. Energy Mater.* 2019, 9, 1901333.
**Rh@N,S-C**	320	Natural seawater	*ACS Sustainable Chem. Eng.* 2019, **7**, 18835.
**Ni_5P_4@$Ni^{2+\delta}O_{\delta}(OH)_{2-\delta}$**	144	Natural seawater	*Appl. Catal. B* 2019, 251, 181.
**Mo_2C-MoP@N,P-C**	346	Natural seawater	*Electrochim. Acta* 2018, 281, 710.
**Mn-NiO-Ni/Ni**	170	Natural seawater	*Energy Environ. Sci.* 2018, 11, 1898.
**2.4% Pt@mh-3D MXene**	280	Natural seawater	*Adv. Funct. Mater.* 2020, 30, 1910028
**NiRuIr-G**	200	Natural seawater	*Electrochem. Commun.,* 2020, 111, 106647
**NiCoN|NixP|NiCoN**	165	Natural seawater	*ACS Energy Lett.* 2020, 5, 8, 2681
**NiCoP/NF**	287	Natural seawater	*ACS Appl. Energy Mater.* 2019, 2, 5, 3910
**h-MoN@BNCNT**	160	Natural seawater	*Adv. Funct. Mater.* 2019, 29, 1805893
**CoMoP@C**	448	Natural seawater	*Energy Environ. Sci.,*2017, 10, 788
**Mo_5N_6**	257	Natural seawater	*ACS Nano 2018*, 12, 12, 12761

3. Supplementary References

1. Panagiotopoulou, P. & Kondarides, D. I. Effects of alkali additives on the physicochemical characteristics and chemisorptive properties of Pt/TiO_2 catalysts. *J. Catal.* **260**, 141-149 (2008).

2. Li, W. et al. Skeletal isomerization of n-pentane: a comparative study on catalytic properties of Pt/WO_x-ZrO_2 and Pt/ZSM-22. *Appl. Catal. A-Gen* **537**, 59-65 (2017).

3. Dolsiririttigul, N. et al. Structure-Activity Relationships of Pt-WO_x/Al_2O_3 Prepared with Different W Contents and Pretreatment Conditions for Glycerol Conversion to 1, 3-Propanediol. *Top. Catal.* **66**, 205-222 (2023).

4. Yu, L. et al.. Non-noble metal-nitride based electrocatalysts for high-performance alkaline seawater electrolysis. *Nat. Commun.* **10**, 5106 (2019).
