# Supplementary material for: Tailoring a local acid-like microenvironment for efficient neutral hydrogen evolution
Source: Nat Commun. 2023 Jul 14;14:4209. doi: 10.1038/s41467-023-39963-8 (PMC10349089; doi:10.1038/s41467-023-39963-8)
Supplement: Supplementary file 3 — Description of Additional Supplementary Files [file 41467_2023_39963_MOESM3_ESM.pdf]

<u>Description of Additional Supplementary Files</u>

Supplementary Movie 1: The colors changing of WO_3 working electrode during the hydrogen insertion.

Supplementary Movie 2: The H_2 bubbles desorption behavior of Pt/C working electrode at 100 mA cm^{-2}.

Supplementary Movie 3: The H_2 bubbles desorption behavior of Ir-H_xWO_3 working electrode at 100 mA cm^{-2}.

Supplementary Movie 4: Changes of H_2 bubbles on Pt/C catalyst surface before and after removing bias.

Supplementary Movie 5: Changes of H_2 bubbles on Ir-H_xWO_3 catalyst surface before and after removing bias.
